# Bacterial Adaptation through Loss of Function

**DOI:** 10.1371/journal.pgen.1003617

**Published:** 2013-07-11

**Authors:** Alison K. Hottes, Lydia Freddolino, Anupama Khare, Zachary N. Donnell, Julia C. Liu, Saeed Tavazoie

**Affiliations:** 1 Lewis-Sigler Institute for Integrative Genomics, Princeton University, Princeton, New Jersey, United States of America; 2 Joint Centers for Systems Biology, Columbia University, New York, New York, United States of America; 3 Department of Biochemistry and Molecular Biology, Columbia University, New York, New York, United States of America; Université Paris Descartes, INSERM U1001, France

## Abstract

The metabolic capabilities and regulatory networks of bacteria have been optimized by evolution in response to selective pressures present in each species' native ecological niche. In a new environment, however, the same bacteria may grow poorly due to regulatory constraints or biochemical deficiencies. Adaptation to such conditions can proceed through the acquisition of new cellular functionality due to gain of function mutations or via modulation of cellular networks. Using selection experiments on transposon-mutagenized libraries of bacteria, we illustrate that even under conditions of extreme nutrient limitation, substantial adaptation can be achieved solely through loss of function mutations, which rewire the metabolism of the cell without gain of enzymatic or sensory function. A systematic analysis of similar experiments under more than 100 conditions reveals that adaptive loss of function mutations exist for many environmental challenges. Drawing on a wealth of examples from published articles, we detail the range of mechanisms through which loss-of-function mutations can generate such beneficial regulatory changes, without the need for rare, specific mutations to fine-tune enzymatic activities or network connections. The high rate at which loss-of-function mutations occur suggests that null mutations play an underappreciated role in the early stages of adaption of bacterial populations to new environments.

## Author Summary

When bacteria encounter a new challenge in their environment, such as treatment with an antibiotic or a poor nutrient source, their population faces tremendous selective pressure to evolve in order to grow better under the new conditions. We typically think of bacterial evolution in terms of what is gained: a bacterium might, for example, acquire an antibiotic resistance gene, or modify an existing enzyme to make better use of a nutrient source. By analyzing the fitness of bacterial populations under more than 100 different conditions, we show that in fact what they lose can be equally important: by rewiring the cell's metabolism, loss of function mutations can provide substantial fitness benefits under many challenging conditions, even cases such as exotic nutrient combinations where some new enzymatic function might seem to be required. Loss of function mutations occur at a much higher frequency than gains of specific functionality due to the larger mutational target area available. The combination of the rapid acquisition and broad functionality of loss-of-function mutations suggests that they play a major role in the early adaptation of bacterial populations to new challenges.

## Introduction

Bacteria evolve to exploit the temporal and spatial structure of their native environments, mapping commonly occurring patterns of stimuli to high-fitness responses [1], [2]. Adaptation occurs through both the acquisition of requisite biochemical and biophysical functions, such as enzymatic capabilities and membrane properties, and evolution of a regulatory network that responds to the environment by deploying the organism's phenotypic capacities in a context-appropriate fashion.

**Figure 1:**
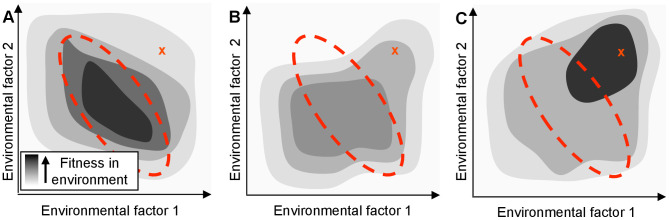
A regulatory network adapted for an organism's native habitat may perform poorly in a new environment. The hypothetical organism's fitness (shading) depends only on the concentration of two environmental factors. The area enclosed by the red dotted line indicates the typical range of these parameters in the native environment. ‘X’ indicates the parameter values in a new environment. (**A**) Fitness of the wild-type organism, which is tuned to be optimal in the native environment. Even if an organism's genome encodes proteins that would confer high fitness in a new environment, its regulatory network might limit the actual fitness achieved. (**B**) Fitness of a mutated network that might result from a single regulatory null mutation. While not optimal, the mutated network may be advantageous in a new environment by breaking the previous mapping of environment to phenotype. (**C**) Extended evolution in the new environment will rewire the organism's regulatory network to allow the cell to optimize the use of its genetic resources (even in the absence of new genes).

In principle, bacteria may grow poorly in a new environment because they lack necessary biochemical capabilities and biophysical properties, or because they express these existing capacities inappropriately. In the former case, mutations that tinker with coding regions to refine existing functions [3]–[5], horizontal gene transfers that introduce novel functions [6], and gene amplifications that enable subsequent neofunctionalization [7] could generate the missing functionality. In the latter case, a bacterium's genome encodes the requisite biochemical and biophysical functions, but the organism's sensory and regulatory networks do not express the functions in a context-appropriate fashion (Fig. 1A). While rare mutations that modulate specific network connections can engender the appropriate regulatory capacity (for example, the hijacking of an aerobic promoter to enable aerobic citrate metabolism in *Escherichia coli* during a long term evolution experiment [8]), comparatively common loss-of-function (null) mutations [9] that produce less specific perturbations could also generate advantageous network adjustments.

The maladaptive properties of null mutations, including their contributions to genome decay are well known [10]. Unlike the rare, specific changes associated with gain-of-function mutations, however, the loss-of-function mutational space can be explored rapidly by an evolving population due to the large number and variety of sequence-level mutations that can give rise to such changes. Although adaptive null mutations have been observed in bacterial laboratory evolution experiments (*e.g.*, [11], [12]), the general potential for null mutations to shape the path of bacterial evolution has not been systematically investigated, despite their potential to enhance fitness by re-deploying the existing capabilities of cells (Fig. 1B). In the discussion below, we refer to any effect in which a single mutation alters cellular fitness by causing non-local changes in information flow or metabolite flux as ‘rewiring’; by definition, any beneficial null mutation which does not exert its impact by removing an actively deleterious reaction must be acting through rewiring.

As we show below, the reconfiguration of cellular metabolism triggered by even one or two such changes often yields improvements in fitness. Rather than provide the cells with qualitatively new capabilities, these mutations improve the cells' application of existing metabolic capabilities to the selective conditions that they are experiencing. While a series of null mutations is unlikely to yield optimal deployment of a cell's constituent genes under novel conditions, loss-of-function mutations can allow the survival and growth of partially adapted individuals that might then further evolve and adapt to the new surroundings (Fig. 1C). Null mutations can also provide access to alternate evolutionary trajectories via different epistatic interactions [13], further expanding the range of phenotypes accessible to an evolving population.

Over the past several decades, numerous detailed studies of specific individual mutants, as well as high-throughput studies of deletion libraries in both bacteria [14] and yeast [15], have identified diverse examples of null mutations that provide a fitness advantage under a wide range of natural and artificial conditions (specific examples in bacteria are listed in Table S1).

**Figure 2:**
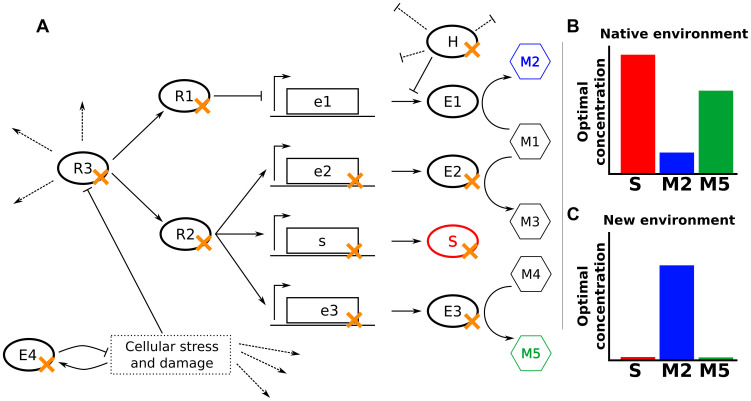
Null mutations increase fitness through varied mechanisms. (**A**) In a hypothetical cellular network, E1–E4 are enzymes, M1–M5 are metabolites, S is a structural protein, R1–R3 are regulatory proteins, and H is a housekeeping protein that inhibits translation and promotes degradation of some mRNAs. Dotted lines indicate other activities of the indicated proteins. The fitness of cells depends only on the levels of S, M2, and M5. (**B,C**) Optimal concentrations of S, M2, and M5 in the native environment (**B**) and a novel environment to which the cells might need to adapt (**C**). Null mutations adaptive in the novel environment are marked in panel (**A**) with an orange ‘x’.

Such beneficial loss-of-function mutations can have varied functional consequences (summarized below in the context of the highly schematized cellular network depicted in Figure 2).

The most obvious mechanism for a beneficial null mutation is to remove a protein or enzyme directly detrimental in the environment of interest (S or E3 in Fig. 2). For example, deletion of *ompF* reduces tetracycline entry into the cell, increasing tetracycline tolerance [16]. Similarly, deletion of the peptidoglycan-recycling enzyme *slt* enhances ethanol tolerance by altering cell wall structure [17].

Many gene products whose deletion is beneficial, however, act multiple steps away from the key cellular property that their deletion modulates. For example, deletion of an enzyme (E2 in Fig. 2) or an upstream regulator (R1 in Fig. 2) may modify metabolic flux to better fit the studied environment. This is illustrated by the combined deletion of *fnr*, *arcA*, and *cafA*, which enhances ethanol tolerance in *E. coli* by increasing ethanol breakdown and subsequent assimilation [17]. Similarly, removal of proteins involved in catabolism or oxidative respiration increases resistance to bactericidal antibiotics by ultimately reducing the production of harmful hydroxyl radicals [18]–[20].

A cellular network's underlying modularity often enables a single regulatory deletion (R2 or R3 in Fig. 2) to alter the levels of multiple components coherently. For example, mutations in many signaling pathways feeding into *flhDC*, the master regulator of flagellar biogenesis in *E. coli*, can modulate flagella-based cellular motility, such as deletion of *ompR* or *envZ* enhancing motility in high-salt conditions [21]. Similarly, the high connectivity of housekeeping genes (H in Fig. 2) in the cellular network can allow their removal to trigger beneficial phenotypes under diverse environments, such as the deletion of Lon protease conferring an advantage in the presence of A22, β-lactams, and ammonium chloride [14], [18]. Additionally, null or hypomorphic alleles of a housekeeping gene can move a cell to a radically different part of the fitness landscape, where epistatic effects can allow accumulation of favorable secondary mutations [13], [22], [23].

The key thread uniting these examples, and the broader array of cases presented in Table S1, is that by altering gene expression and the flow of metabolites, loss-of-function mutations trigger far reaching changes in the cell's regulation and metabolism. As detailed in the meta-analysis presented below, these changes frequently prove adaptive under novel environments. The relative abundance of null mutations coupled with their adaptive potential suggests that specific null mutations likely represent common early steps in the evolution of bacterial populations encountering a new environment.

Here, we examine comprehensively the potential of loss-of-function mutations for adaptation to novel environments. We first use a meta-analysis of genome-wide fitness data from transposon-insertion and in-frame deletion mutations across 144 conditions from 7 studies (including new findings described below) to show that adaptive null mutations are extremely abundant and disproportionately affect enzymatic and regulatory pathways. We then take as a case study the fitness profile of populations of *E. coli* transposon-insertion mutants in a set of unusual, nutrient-limited environments. The transposon insertions provide a convenient method to generate tagged null mutations that can be easily identified on a genome-wide scale and are likely to reflect phenotypes arising from common indels and point mutations that result in loss-of-function. In our media challenges, single loss-of-function mutations are sufficient to increase the growth rate up to twofold, demonstrating the suboptimal utilization of existing capacities by the wild-type strain and the ease with which null mutations can enhance fitness through metabolic and regulatory network rewiring.

## Results

### Beneficial null mutations preferentially target enzymatic and regulatory functions

Cases of beneficial null mutations have been noted previously in a wide variety of studies of both laboratory-evolved and wild strains; many of the best-characterized examples are summarized in Table S1. Any such list, however, is biased by the limited set of conditions and mutants that have been characterized in detail. The increasing availability of quantitative fitness data from genome-wide screens of loss-of-function mutants in a wide variety of conditions allowed us to systematically study the adaptive potential of null mutations at a much more comprehensive scale.

We performed a meta-analysis of null mutation fitness data from a total of 144 conditions from 7 studies in *E. coli* MG1655 and BW25113 (including new data described below). For each condition, we identified genes for which null mutations gave significant increases or decreases in fitness and then examined the complete data set for evidence of over-representation of specific biological functions (see Materials and Methods for details on the data sets, which included experiments from both in-frame deletions and transposon-mutagenized libraries, and statistical processing).

**Figure 3:**
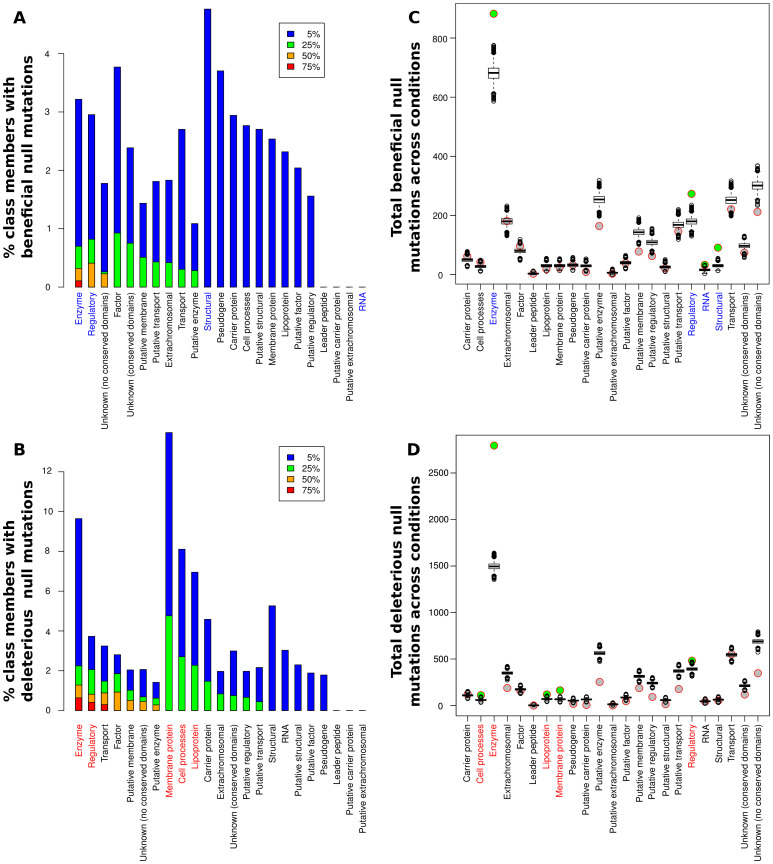
Contributions of null mutations to fitness by functional category. (**A,B**) Percentage of members of each class for which null mutations caused significantly beneficial (**A**) or deleterious (**B**) fitness effects in at least 5%, 25%, 50%, and 75% of the 144 conditions analyzed. Colored labels on the x-axis indicate classes with a significant enrichment in null mutations (q<0.01 by a resampling test; see main text and Table S2). (**C,D**) Circles: total number of significant beneficial (**C**) or deleterious (**D**) null mutations present in each class across all conditions in our meta-analysis. Box plots: simulated null distribution of the same statistic for each class (see main text and Materials and Methods for details). Classes showing significant enrichments (q<0.01) relative to the corresponding null distribution have their label colored on the x-axis and the circle representing their observed value filled in green. No bar appears for the RNA class in panel A because fewer than 5% of the conditions had beneficial nulls from the RNA class; see main text for details. All classifications are from GenProtEC (http://genprotec.mbl.edu/files/geneproductfunctions.txt).

While the relative portions of each functional class showing significant fitness effects (positive or negative) upon deletion varied greatly among the conditions (see Fig. S1), some clear trends were present. Overall, at least one beneficial null mutation was identified in all but five of the 144 conditions considered. In particular, we found adaptive (and maladaptive) deletions of regulatory proteins and enzymes in over half of the experimental conditions assayed (Fig. 3A,B), while significant contributions from other classes were generally less frequent. For a more quantitative assessment, we used a resampling approach to determine the significance of each category's contribution to the observed fitness changes relative to its size (Fig. 3C,D and Table S2). Only enzymes and regulatory proteins showed enrichments of null mutations that both raise and lower fitness. Structural proteins and RNA genes also contained significant numbers of beneficial deletions, mainly due to a large number of beneficial deletions from those classes present in a small number of experimental conditions (we could not, however, detect a notable unifying factor in the conditions under which null mutations in these classes of genes were beneficial). The RNA case in particular is dominated by two conditions under which transposon insertions in ribosomal RNAs were beneficial, and thus must be viewed with some caution. On the other side, membrane proteins, lipoproteins, and cell process proteins also contributed higher than expected numbers of deleterious null mutations, although their contributions were still lower than the frequency for enzymes or regulatory proteins. The abundance of adaptive regulatory null mutations was perhaps our most striking finding; the fact that purely regulatory mutations can allow bacteria to adapt to a wide variety of extreme conditions illustrates the extent to which the physiological capabilities of microbes exceed their regulatory logic, and the relative ease with which knockouts of appropriate regulators can rapidly rewire a maladaptive regulatory network. It was also instructive to consider the breadth of conditions under which a given null mutation could be adaptive; the set of genes for which null mutations were beneficial in at least 10 conditions in our meta-analysis is shown in Table S3. Consistent with the above findings, 6 out of 7 such genes coded for either enzymes or regulatory proteins, and housekeeping genes (*lon, dnaJ*) played a particularly prominent role. It is likely that these null mutations, as well as loss of function of the nucleoid-associated protein *fis*, exert their widespread beneficial effects by globally altering expression of other genes, similar to the mechanism of action of a recently characterized *rho* hypomorph that proved beneficial in more than ten different conditions [23].

### Abundance of strongly beneficial null mutations under severe nutrient deprivation

**Figure 4:**
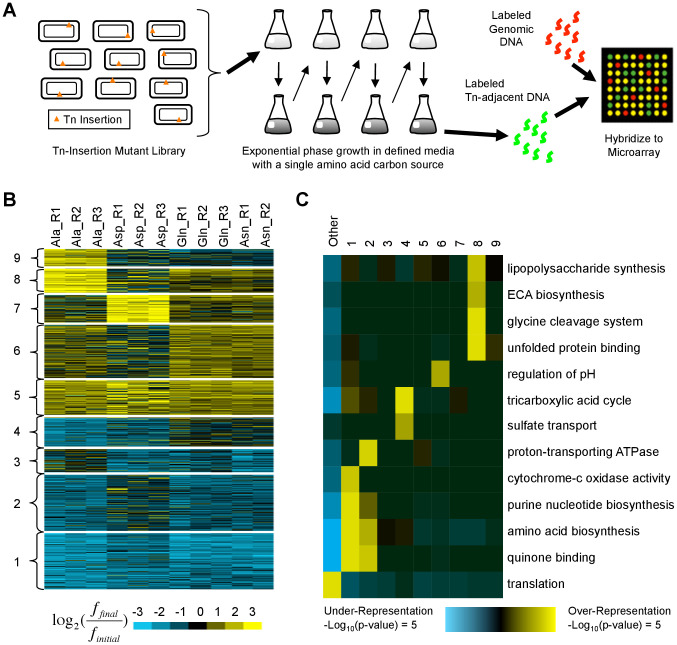
Identification and characterization of transposon insertion locations that alter fitness in single amino acid media. (**A**) A library of ∼500,000 independent transposon insertion mutants [21] was grown for ∼20 generations in defined media with a single amino acid carbon source. Serial dilutions were used to keep the cultures in exponential phase. To characterize the distribution of transposon insertion locations in the population, DNA adjacent to the transposons was amplified, labeled, and hybridized to a custom ORF microarray. (**B**) K-means clustering was used to organize the fitness profiles of 809 genes in whose vicinity transposon insertions significantly altered fitness in at least one media (see Materials and Methods). Each row represents a gene; each column contains data from a different biological replicate. Values compare the fraction of mutants with transposon insertions in or near each gene before (*f_initial_*) and after (*f_final_*) growth in single amino acid media. Yellow (blue) indicates an increase (decrease). (**C**) Shown are functional enrichments and depletions based on Gene Ontology annotations that iPAGE [24] identified in the clusters from (**B**) and the genes not in any cluster (‘Other’). ECA: enterobacterial common antigen.

The previously published data sets analyzed above consist primarily (although not entirely) of chemical or physical hazards added to otherwise standard growth media. An equally realistic scenario for a microbe is to encounter nutrients that the organism's metabolism is poorly equipped to utilize. The relative roles of regulatory rewiring vs. acquisition of new functions in adaptation to such conditions and the potential for adaptive null mutations in these cases remain largely unexplored. To further understand the potential for null mutations to alter fitness in the face of a metabolically challenging environment and to explore the mechanisms employed, we propagated a library of *E. coli* MG1655 transposon-insertion mutants [21] in four media conditions where the parental strain grew poorly (defined M9 media with alanine, glutamine, aspartic acid, or asparagine as the sole carbon source; see Fig. 4A). In addition to including the data in our meta-analysis, we identified the 809 insertion locations that caused the greatest increases and decreases in fitness (Fig. 4B) (see Materials and Methods and Dataset S1). The use of a transposon library, containing ∼10^6^ disruptive perturbations, allowed us to explore the space of possible adaptive null mutations more rapidly and comprehensively than evolutionary approaches. Although such mutations are unlikely to be found in the wild, the resulting phenotypes mirror those of common point mutations or small insertions and deletions that cause loss of function.

Using the pathway analysis tool iPAGE [24], we found that clusters of genes whose disruption was deleterious (clusters 1–4) are enriched for genes whose products participate in nucleotide and amino acid biosynthesis, functions essential in the growth media we used (Fig. 4B,C). In contrast, the clusters containing beneficial insertion locations (clusters 5–9) showed varied and generally weak functional enrichments, suggesting that alterations to many distinct pathways can increase fitness.

**Figure 5:**
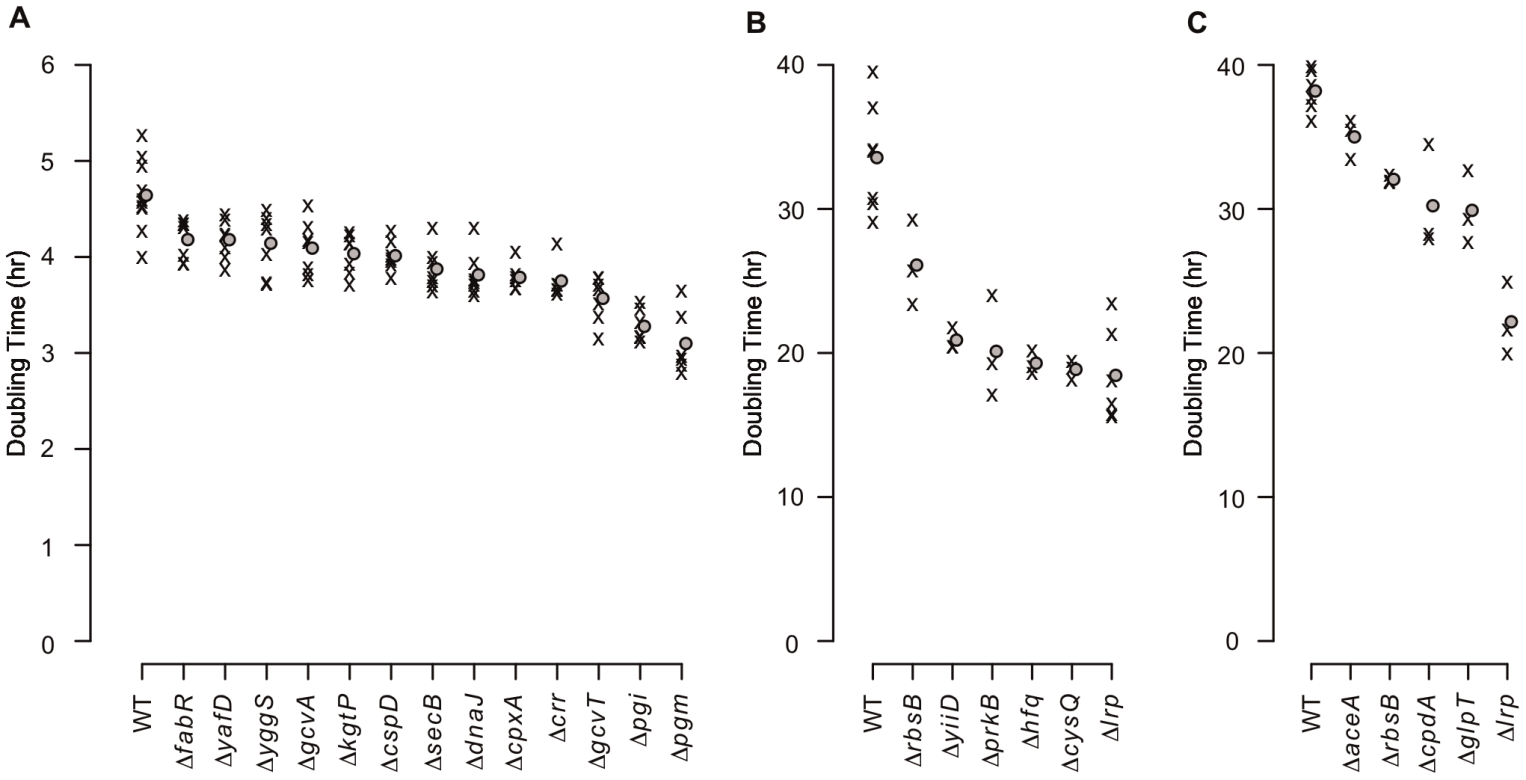
Growth rates of mutants with in-frame deletions. Average exponential phase doubling times in defined media with (**A**) alanine, (**B**) glutamine, or (**C**) asparagine as the sole carbon source. ‘X’s denote individual measurements. Circles indicate mean doubling times. Of the 48 growth tests performed as a result of the transposon enrichment experiments (24 for alanine, 11 for glutamine, and 13 for asparagine), only the 24 strain/media combinations that grew significantly faster than the parental strain are shown (1-sided Mann-Whitney test, significance cutoff of 5% false discovery rate (FDR) for the entire dataset). Significant q-values are in Table S5. WT: wild-type.

As transposon insertions do not necessarily cause a null phenotype [21], we tested in-frame deletions for a representative set of candidate genes in three of the growth conditions (Table S4). As expected, many of the null mutants grew significantly faster than the parental strain in the experimental media (Fig. 5 and Table S5). Doubling times dropped by as much as 30% for alanine media and nearly 50% for each of glutamine and asparagine media – a substantial fitness increase – showing how poorly the parental strain utilizes its existing capacities in these extreme environments.

Transcriptome analyses of four fitter-than-wildtype mutants in each of alanine and glutamine media (Dataset S2) revealed that each mutant had a distinct expression pattern. While overlaps among the genes up- and down-regulated in individual mutants were generally larger than would be expected by chance (Table S6), the number of genes whose expression exhibited large (>2-fold) changes (Fig. S2A,B) and the functional categories overrepresented among the differentially expressed genes varied widely among the mutants (Fig. S2C–F). In particular, expression differences among chemotaxis and flagellar biosynthesis genes were especially prominent (Fig. S3). The diversity of transcriptome changes with a net beneficial effect illustrates the non-optimality of the wild-type genetic network in the experimental media and the varied possibilities for improvement. Additionally, the breadth of transcriptome changes in the Δ*pgi* and Δ*cysQ* strains (Fig. S2A,B) demonstrates the potential for enzymatic null mutations to rewire a large portion of the cell's regulatory and metabolic network.

**Figure 6:**
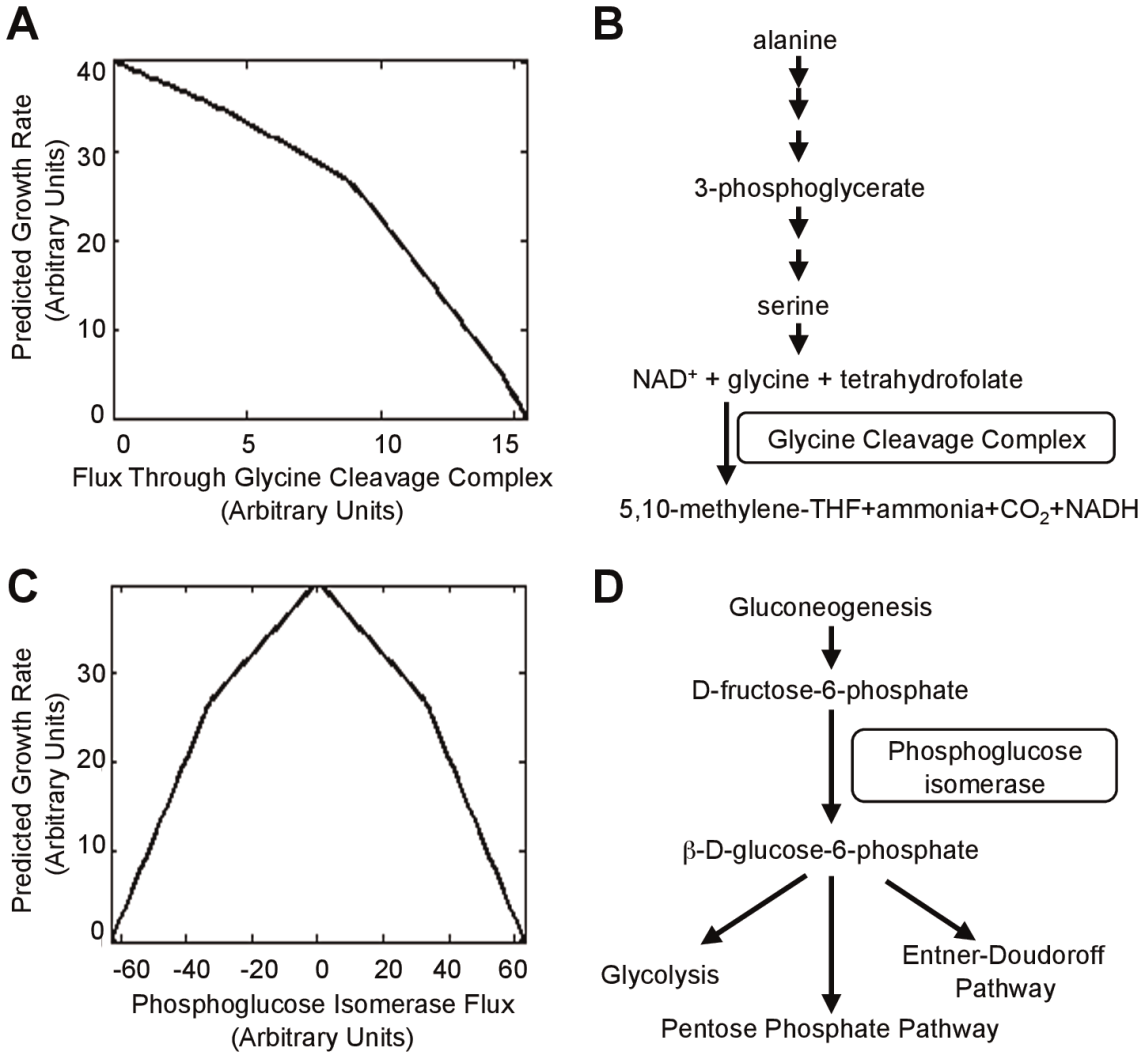
Deletions that move a cell towards the theoretically optimum flux distribution. (**A**) Maximum growth rate of the iAF1260 flux balance analysis (FBA) model [27] on alanine as the sole carbon source when flux through the glycine cleavage complex is set to the indicated value. (**B**) The pathway shows a metabolic flux configuration capable of supporting low-levels of glycine degradation (less than ∼9 units in (**A**)) by the GCC. (**C**) Maximum simulated growth rate when flux through phosphoglucose isomerase is set to the indicated value. Positive fluxes (conversion of glucose to fructose) utilize a cycle where xylose isomerase, which is encoded by b3565, converts fructose back to glucose. To generate negative fluxes (fructose to glucose), the model runs gluconeogenesis and then degrades the glucose through the pentose phosphate pathway. (**D**) The reaction catalyzed by phosphoglucose isomerase connects the gluconeogenesis and glucose degradation pathways.

To better understand the mechanisms by which the null mutations tested above lead to increased fitness, we used flux balance analysis (FBA), which determines in a regulation-independent fashion ways a cell could use its metabolic capabilities to maximize its growth rate in a specific environment [25]. FBA simulations indicated that *E. coli* attains its maximum growth rate in alanine media when the glycine cleavage complex (GCC) is not utilized (Fig. 6A), consistent with our observed benefits of deletion of GCC components (Fig. 5A). The cost of synthesizing increasing amounts of serine only to degrade it to glycine likely accounts for the decreasing growth rate as GCC flux increases (Fig. 6B). Simulations also indicated that phosphoglucose isomerase should be inactive during rapid growth in alanine media because flux through the enzyme creates a futile cycle (Fig. 6C,D); our results validated that prediction (Fig. 5A). Both of these examples illustrate how fitness defects can be caused, not by lack of enzymatic functions, but rather their context-inappropriate utilization.

Overall, flux variability analysis [26] indicated that proteins encoded by ∼860 (numbers range from 858 for glutamine media to 869 for alanine media) of the 1260 genes in the iAF1260 genomic reconstruction for *E. coli* K-12 MG1655 [27] catalyze reactions in pathways that must be ‘off’ to allow maximum growth (see Materials and Methods). Likely a variety of deletions, acting both directly and indirectly, can reduce or eliminate the superfluous fluxes.

Consistent with the results of our meta-analysis above, deletions of regulators also provided substantial fitness advantages; prominent examples are *cpxA* in alanine media (+22% growth rate) and *lrp* in both glutamine (+82% growth rate) and asparagine media (+72% growth rate). It is also useful to note that most of the beneficial mutations studied here are neutral or deleterious in environments other than the one in which they were identified (Fig. S4), consistent with the notion that they introduce specific perturbations that increase cellular fitness in the new environment. Thus, our results indicate that even when faced with an environment that imposes severe metabolic challenges, null mutations can alter the regulatory and metabolic network of bacterial cells to greatly increase fitness without the gain of additional enzymatic functions, supporting our broad hypothesis that null mutations play a substantial role in adapting to diverse novel environments.

## Discussion

Our experimental results and meta-analysis of previous studies demonstrate the substantial potential of loss-of-function (null) mutations to aid in adaptation to novel environments through regulatory and metabolic rewiring. We find that the overarching effect of many null mutations is to improve the match between a cell's regulatory network, which is well-adapted to the organism's native habitat, and the contingencies of the new environment. This is particularly true for deletions of genes in the two functional classes in which we see the most widespread over-representation of beneficial null mutations: enzymes and regulatory proteins (Fig. 3).

Regulatory mutations, especially those in *cis*-regulatory sequences, have long been thought to play an important role in adaptation (reviewed in [5]), and our work shows that null mutations in regulators themselves also make a substantial contribution, increasing and decreasing the activity of cellular modules and facilitating the emergence of new phenotypes. The prevalence of adaptive null mutations in regulators illustrates that the phenotypic capabilities of bacterial cells – that is, the range of environments in which they possess the capacities to thrive – far exceed their regulatory capacity, the range of environments in which they can respond productively. When cells possess the biochemical capabilities for thriving under extreme conditions but fail to deploy those resources due to the constraints of the overlying regulatory network, regulatory mutations can rapidly allow the appropriate expression of those phenotypic capabilities. The GASP (growth advantage in stationary phase) phenotype [28] that arises in very old *E. coli* cultures provides a clear example: prolonged incubation in stationary phase yields cells with mutations that greatly enhance stationary phase fitness, including null mutations in the regulator *lrp* [29] and mutations attenuating activity of the sigma factor *rpoS* [30], [31].

Enzymatic deletions also remodel cellular networks, albeit in a different way. Metabolic engineers are quite aware that well-chosen deletions can boost yields by redirecting fluxes or removing undesirable byproducts [32], [33], and the present work presents multiple examples of the utility of silencing enzymes. Similarly, when a cell's regulatory network erroneously expresses a metabolic pathway, knockouts of one of the component enzymes can often ameliorate the fitness deficit. For example, Bollenbach and coworkers recently found that bacterial growth in the presence of DNA synthesis inhibitors was suboptimal due to overexpression of ribosomal RNA operons under these conditions and could be improved by deletion of most copies of those genes [34].

A beneficial mutation need not cause a large fitness gain to impact the trajectory of an evolving population. Many of the beneficial null mutations discussed here have phenotypic effects, such as approximately twofold changes in antibiotic minimum inhibitory concentration (MIC), smaller than the eventual level of adaptation observed in laboratory-evolved or clinical populations. Nevertheless, a population with even a small advantage under stressful conditions will be favored over time, and the increased growth rate itself will increase the odds (per unit time) of acquiring additional adaptive mutations. Furthermore, even the accumulation of many mutations of individually small effect can give rise to a dramatic phenotypic difference, as has been observed in the case of antibiotic resistance in both laboratory strains [18] and clinical [35] populations. The eventual evolutionary trajectory of the population may include reversion of the original adaptive null mutation, if the bacteria re-encounter conditions where the gene function is beneficial.

Beneficial null mutations also enable rapid fitness increases by presenting a large mutational target size. While both null mutations and the acquisition of novel protein functions can cause widespread alterations to cellular phenotypes, the comparatively higher probability with which null mutations occur amplifies their importance in adaptive evolution. In contrast with gain of function mutations that require one of a few specific changes to a protein or regulatory element, loss of function mutations can arise from any frameshift, nonsense mutation, or insertion in a coding region if it occurs early enough in an ORF, as well as through a variety of missense mutations specific to any given protein.

We very conservatively estimate that null alleles arise at a rate on the order of 10^−8^ per gene per cell division (assuming the mutation rate is on the order of 10^−10^ per nucleotide [36], null alleles arise only from a nonsense mutation in the first half of an ORF, the average gene length is 1 kb, and codon usage is uniformly distributed). Bacteria also carry genetic programs for generating additional diversity under stress through error-prone DNA repair pathways [37], [38], likely making it even easier for cells to acquire adaptive null mutations through the generation of frameshift or missense mutations. Genomic rearrangements mediated by insertion elements can likewise further accelerate creation of loss of function mutations. For example, beneficial loss of function of the *rbs* operon has been observed to arise at a frequency of 5 * 10^−5^ per generation in laboratory evolution experiments due to the operon's proximity to an IS150 element [39]. The combination of the rate at which null mutations arise and the breadth of circumstances under which these mutations can be beneficial may be at least partly responsible for the observation that *E. coli* acquire small beneficial mutations (∼1% change in fitness) at a surprisingly high rate of about 10^−5^ per generation [40]. Consistently, beneficial null mutations have frequently been shown to make substantial contributions to fitness in laboratory evolution experiments [11], [12] and in a wide variety of natural conditions (reviewed in Table S1 and in the examples below).

Most of the beneficial null mutations studied here were identified in a single culture condition (albeit with the usual fluctuations in media composition that occur with cell growth in batch culture); it is likely that adaptation to novel natural environments involves an even more complex interplay of physicochemical parameters, where antagonistic pleiotropy may reduce the adaptive potential of single null mutations. However, far from being laboratory artifacts, adaptive null mutations are being increasingly recognized in natural and clinical settings as well. For example, null mutation-mediated adaptation contributed to the divergence of *Bacillus anthracis* from a *Bacillus cereus* ancestor. In addition to two virulence-factor encoding plasmids (pXO1 and pXO2), *B. anthracis* is characterized by a specific and ubiquitous nonsense mutation in *plcR*, which encodes a pleiotropic transcriptional activator [41], [42]. The *plcR* null mutation in *B. anthracis* leads to significant reduction in the secretion of several degradative enzymes and virulence factors [43]. Although conflicting reports exist about the evolutionary pressures underlying the selection of this null mutation [43], [44], the current hypothesis is that *plcR* inactivation is part of the co-evolution of the chromosome and the pXO1 and pXO2 plasmids that led to the emergence of *B. anthracis* as a separate species [42].

The evolution of pathogenic *Shigella* strains from their *E. coli* ancestors was also mediated by null mutations in several anti-virulence genes, in addition to the acquisition of pathogenicity islands and a virulence plasmid [45], [46]. Deletion of the *cadA* gene and null mutations in the *nadA* and *nadB* genes in the *Shigella* genome prevent the formation of cadaverine and quinolinate respectively, and both these molecules inhibit multiple aspects of *Shigella* pathogenicity [47]–[49]. Similarly, null mutations in *speG* allow the accumulation of spermidine, which increases *Shigella* resistance to oxidative stress and survival within macrophages [50].

Beneficial null mutations not only aid in the evolution of new species of pathogens, but can also facilitate the repeated adaptation of infecting pathogens to specific host niches. For example, null mutations in key regulators mediate adaptive diversification of *Pseudomonas aeruginosa* during chronic lung infections in cystic fibrosis patients, leading to non-piliation, flagellum loss, lack of quorum-sensing, and mucoidity from increased alginate production [51]. The most common cause of the switch to mucoidity is loss of *mucA*, which encodes an anti-sigma factor that sequesters AlgT, an activator of alginate biosynthetic genes [52]. Loss-of-function mutations in *lasR*, which encodes a transcriptional regulator, are frequently seen in isolates from the cystic fibrosis lung and lead to quorum-sensing-negative phenotypes and reduced expression of virulence factors [53]. The phenotypes resulting from these deletions are within the physiological capabilities of the *P. aeruginosa* genome but are normally repressed by the regulatory network. Null mutations in important regulators alter the expression of entire modules and rewire the network to enable *P. aeruginosa* to adapt from its original niches as a free-living organism and acute infectious agent to long-term survival as a chronic infection in a host, although this adaptation may be important only for the specific infecting population and not for the species at large. Improved understanding of the contributions of null mutations to fitness is thus crucial for elucidating the evolutionary paths taken by evolving bacterial populations.

These findings might also facilitate progress on other challenges such as understanding bacterial adaptation during chronic infections, engineering bacteria for introduction into novel environments or microbial communities, and culturing ‘unculturable’ bacteria [54]. Such ‘unculturable’ species might possess all of the biochemical capabilities necessary to grow in monoculture on common cultivation media, but simply not utilize them properly in an environment so different from their native habitat, leading to an adaptation barrier to lab conditions. Culturing such bacteria may thus require more sophisticated interventions than simple supplementation with additional nutrients.

## Materials and Methods

### Strains and growth conditions

Unless otherwise noted, media was M9 [55] lacking NaCl (48 mM Na_2_HPO_4_, 22 mM KH_2_PO_4_, 19 mM NH_4_Cl, 2 mM MgSO_4_, 0.1 mM CaCl_2_, and 10 µM thiamine), supplemented with 2 g/L of the carbon source and micronutrients [56] at the following final concentrations: 3 nM (NH_4_)_6_(Mo_7_O_24_), 400 nM H_3_BO_3_, 30 nM CoCl_2_, 10 nM CuSO_4_, 80 nM MnCl_2_, and 10 nM ZnSO_4_. No supplementary iron source was added. LB media was 1% Bacto Tryptone, 0.5% yeast extract, and 0.5% NaCl. Due to glutamine's limited stability in solution, we prepared glutamine media fresh for each experiment. Media used for growth curves with glucose included 0.01% Tween-20 to eliminate optical artifacts due to biofilm formation [23]. Unless otherwise noted, we grew cell cultures at 37°C and shook them at 250 rpm.

To make clean, in-frame deletions, we transduced KanR (kanamycin resistance cassette) marked alleles from the Keio collection [57] into strain AH28 (MG1655 *ΔlacZ*) using P1*vir* phage [58] and removed the markers using a FLP recombinase system [59]. We confirmed each mutant's identity by comparing sizes of PCR products of the region containing the putative gene deletion in the mutant and parental strains. Table S4 lists all strains used in this work.

### Competitive enrichments and genetic footprinting

Before starting the single amino acid cultures, we grew thawed aliquots from the transposon library [21] in LB for three generations and washed the cells in M9 salts lacking a carbon source. Next, we added ∼10^8^ cells to 5 ml of M9 media with the appropriate amino acid as the sole carbon source. Using serial transfers, we maintained the cultures in exponential phase above a minimum population size of ∼10^8^. To reduce the impact of spontaneous mutations while allowing for the detection of subtle fitness effects, we harvested and analyzed cultures after twenty generations [60]. We carried out transposon footprinting as described previously [21].

### Determining significant transposon insertion locations

Data (ratios of transposon signal to genomic DNA signal) were sum-normalized and then log-transformed (base 2) to give increases and decreases similar magnitudes. Arrays were normalized to the mean of five hybridizations of the transposon library prior to selection [21] by fitting a loess [61] curve (with the span parameter set to 0.3) to the intensities on the experimental array as a function of the mean intensities for the same genes on the reference arrays, and then subtracting from each gene the loess-predicted value. After normalization, transposon insertion locations that did not change in abundance in response to growth in single amino acid media should be distributed around zero. As a summary statistic for each gene in a given condition, we used the value closest to zero if the normalized values from all replicates had the same sign and zero if they did not.

To evaluate the significance of the summary statistics, we constructed a separate null distribution of 500,000 “genes” for each of the four amino acids. Each gene contained either three (for alanine, aspartic acid, or glutamine) or two (for asparagine) data points. Samples for each gene came from a t-distribution with 4 degrees of freedom, with standard deviation equal to the standard deviation of the normalized experimental samples of a randomly chosen gene for the amino acid of interest and mean set to the median of the five data points for a (possibly different) randomly chosen gene from the normalized, unselected hybridizations. Summary statistics were calculated for the null distribution as they were for the data, and gene level p-values were set to the fraction of null genes with summary statistics exceeding the actual observed value in magnitude. We chose the significance cutoff for each amino acid separately to give an estimated 5% FDR.

We excluded genes that the Profiling of *E. coli* Chromosome database version 4 marked as essential (http://www.shigen.nig.ac.jp/ecoli/pec/index.jsp) [62]. Of the 3792 genes tested for significance, 809 were significant in at least one condition.

Expression profiles were subjected to k-means clustering using Euclidean distance as the distance metric. For each gene, we included the expression level in each biological replicate as well as the average across replicates for each condition. During clustering, we assigned columns of averages ten times the weight of columns of individual biological replicates. For visualization purposes, enrichment values were restricted to the range between −3 and 3, and extreme values are shown as either −3 or 3.

### FBA simulations

FBA simulations used the iAF1260 genomic reconstruction for *E. coli* K-12 MG1655 [27] in MATLAB with SBML and COBRA toolboxes [26]. Simulations were done in computational minimal media [27] with the sole carbon source set to 10 mmol g DW^−1^h^−1^ with the Ec_biomass_iAF1260_core_59p81M biomass objective function. A gene was deemed non-essential for maximum growth in a medium if simulation of the full model and the model lacking that gene gave the same growth rate.

We used Flux Variability Analysis [26] to identify fluxes that needed to be zero to obtain the maximum growth rate. Then, all non-essential genes that either by themselves or in combination with other genes directly catalyzed those reactions were considered to be in a pathway that needed to be zero for maximum growth. Due to numerical noise, fluxes were not required to be exactly zero; changing the thresholds did not alter the results qualitatively.

### Growth curves

All growth curves in 96-well plates used flat-bottom, untreated, polystyrene plates (Corning #3370) with 150 µl of media per well. To reduce evaporation, we covered samples with 100 µl mineral oil [63]. A SynergyMx (Biotek; Winooski, VT) read the absorbance at 600 nm. We subtracted the absorbance of wells with media and oil but no cells from all readings as background. Unless otherwise specified, the reader shook the plates continuously on its ‘medium’ setting and maintained the temperature at 37°C.

For growth curves in glucose media, we grew most strains overnight in the test media and diluted 375-fold into fresh media. Due to their slow growth rate on glucose, we grew strains ZD8, Z18, ZD56, ZD59, and ZD60 overnight in glucose media supplemented with alanine, proline, and asparagine (0.5 g/L each) and then washed them before final dilution into glucose media. We measured absorbance every 8 minutes for 36 hr and calculated growth rates as the least squares fit to the logarithm of the part of the background-corrected growth curve between 0.015625 and 0.0625 (before taking the logarithm). Most strains doubled at least three times before reaching the target absorbance range. For the remaining strains, we identified the exponential growth region by hand and adjusted the target range as necessary. The r^2^ value of each fit was required to be greater than 0.99.

To determine doubling times in alanine media, we grew cultures overnight in LB, washed them, and diluted them 300-fold into media in 96-well plates. We shook plates at 250 rpm in an incubator and measured absorbance several times a day starting at ∼20 hours after inoculation; we kept cultures in exponential phase (background-corrected absorbance less than 0.15) using 15-fold serial dilutions. The doubling time estimates came from least-squares fits to the logarithm of the background-corrected absorbance readings multiplied by the total dilution prior to the reading. Data for each fit included at least 4 samples (average 11.3) spanning at least 6 generations (average 14.9) and yielded an r^2^ value of at least 0.95.

We determined growth rates in glutamine and asparagine media in two stages. As an initial filter, we attempted to determine growth rates in 96-well plates as was done for alanine media, but the wide range of doubling times resulted in lower quality data than we had obtained in alanine media. Thus, we retested those mutants that exhibited an advantage over the parental strain individually. In this second stage, which was used to generate all data reported for glutamine and asparagine media, we grew strains as 20 ml cultures in 250 ml flasks and shook them at 250 rpm. We removed culture samples several times a day and read the absorbance at 600 nm on an Ultrospec 3100 pro. We started cultures by diluting washed, LB-grown overnight cultures 100-fold into fresh test media, and after ∼2 generations of growth, we diluted cultures a second time. Sampling started after an additional ∼1 generation of growth (∼3 generations total in the test media) when the absorbance reached ∼0.01 and continued until the absorbance exceeded 0.1. We identified the linear portion of the logarithm of each growth curve manually and then subjected it to a linear least-squares fit to determine the doubling time.

### Transcriptional profiling

We washed and diluted LB-grown overnight cultures into glutamine or alanine media. After ∼5 generations of growth, we harvested samples undergoing mid-exponential phase growth and added 2 ml of culture to 4 ml of RNAprotect Bacteria Reagent (Qiagen). We incubated the mixture at room temperature for 5 min and then centrifuged it at 5000 g for 10 min. We removed the supernatant and stored the pellet at −80°C. We isolated RNA using the Norgen Total RNA Purification Kit according to the manufacturer's directions except that in the last step we eluted the RNA in 35 µL of the kit's elution solution. We poly-adenylated the RNA by combining 31 µl RNA (undiluted from the previous step) with 4 µl 10× Poly(A) Polymerase Reaction Buffer (New England Biolabs), 4 µl 10 mM ATP, and 1 µl (5 U) *E. coli* Poly(A) polymerase (New England Biolabs) and incubating at 37°C for 30 minutes. Then, we cleaned samples with an RNeasy Mini Kit (Qiagen) and labeled them with cyanine 3-CTP or cyanine 5-CTP dye using the Low Input Quick Amp Labeling Kit (Agilent) starting with 200 ng of RNA per sample. We labeled strain AH28 with Cyanine 5-CTP and mutants with Cyanine 3-CTP. We then hybridized samples to an Agilent *E. coli* Gene Expression Microarray (8×15K format, Catalog # G4813A-020097) according to the manufacturer's instructions, scanned the resulting slides using a High-Resolution C Scanner (Agilent), and extracted features using Agilent's Feature Extraction Software version 9.5 using protocol GE2-v5_95_Feb07 without spike-in controls. We used the ‘LogRatio’ value in subsequent analyses. We averaged all values for the same ORF and values from the two biological replicates performed for each comparison.

To estimate the false positive rate, we approximated the null distribution by taking the difference of the values from the two biological replicates for the same gene and dividing by two. This produced a data set with a zero mean and the same noise distribution as that produced by averaging. We calculated a single null distribution for all 8 samples (4 mutants in alanine and 4 in glutamine). Then, the chance of a false positive was the number of samples from the null distribution greater than 1 or less than −1 (i.e., a two-fold change). The false discovery rate is the estimated number of false positives divided by the number of genes deemed significant.

For each mutant, we ran iPAGE [24] in discrete mode on three sets of genes: those whose expression increased at least 2-fold between the mutant and the parental strain, those whose expression decreased at least 2-fold, and the remaining genes. We also ran iPAGE in continuous mode with various numbers of bins and identified categories similar to those in Fig. S2.

Expression data are in Dataset S2 and in the Gene Expression Omnibus (accession GSE30345).

### Meta-analysis

We used a total of 144 data sets showing the fitness effects of null mutations in *E. coli* K12 strains; we obtained 113 from the comprehensive characterization of knockout strains (in the BW25113 background) performed by Nichols *et al.* [14], with the remainder coming from a series of experiments on transposon mutagenized libraries (in the closely related MG1655 background) performed by the Tavazoie laboratory [17], [18], [21], [23], [64] including this work. We excluded all genes identified as potentially essential during the construction of a gene-by-gene deletion library in BW25113 [57] or in a series of chromosomal deletions [62] from analysis, as null mutations of essential genes are clearly impossible. In combining the studies, we followed the significance calling metrics of the original authors as closely as possible.

For the datasets from Girgis *et al.* [18] we used the published significance criteria. For data from Freddolino *et al.* [23], we generated a p-value for each gene by resampling the probe level scores from the full genome-wide distribution 10,000 times to create a null distribution, and then applied a 1% FDR for significance calling. Otherwise, for conditions with two or more biological replicates, we determined significance at a FDR of 5% as we did for the single amino acid experiments in this work. The selections from Girgis *et al.* [21] were extremely stringent, making insertions resulting in average and below-average fitness effectively indistinguishable; hence, for those data sets we only included beneficial insertions in the meta-analysis. Amini *et al.*'s [64] data set on biofilm induction by poly-N-acetylglucosamine did not contain any significant genes at a 5% FDR, so we instead marked as significant only the three gene deletions whose phenotypes the work experimentally confirmed.

Similarly, when only a single biological replicate was available for a condition (motility in high-salt media [21] or fitness in various ethanol concentrations [17]), we counted as significant only those deletions whose fitness contributions the studies individually verified. We assembled a single (non-concentration-specific) set of deletions altering fitness in ethanol.

To identify significant deletions in the Nichols *et al.* [14] data set, we retained the authors' normalization (each of 324 experiments individually normalized to zero mean and IQR = 1.35) and the authors' null model (normal distribution with zero mean and standard deviation of one). Then, considering all experiments collectively, we chose a cutoff corresponding to a 5% FDR. Finally, for each series of dosage titrations for a given condition, we used only the data from the highest dose (113 experiments total). We excluded data from strains carrying hypomorph alleles of presumed essential genes.

To assess the significance of the numbers of beneficial or deleterious null mutations of different classes relative to that expected if the class labels were not significant, we performed the following resampling test: for each gene class/condition combination, we generated simulated distributions with the same total number of elements as the number of genes considered from that class in the corresponding condition in the real data, with the probability of each element being ‘true’ (that is, beneficial or deleterious) equal to the average probability of a gene being beneficial or deleterious (as appropriate) across all genes under that condition. For each gene class, we then took the sum of ‘true’ elements across all conditions as a summary statistic. The (one-tailed) p-value for enrichment of beneficial (or deleterious) genes in each class is obtained by comparing the observed number of beneficial (or deleterious) genes in that class to 10,000 simulated draws for the same class; the p-value is the fraction of those simulated draws which yield a summary statistic greater than or equal to the observed value. Significance of these classes was then determined by applying the Benjamini-Hochberg procedure [65] to the raw p-values, to identify classes that were significant at an FDR of 0.01. The resampling procedure described here yields the distribution shown in Figure 3CD and the q-values in Table S2.

### Data availability

Expression Data has been uploaded to the Gene Expression Omnibus (GEO) (accession GSE30345).

## Supporting Information

Dataset S1 Transposon-footprinting data from competitive enrichments.
(XLS)

Dataset S2 Data from transcriptional profiling of mutants.
(XLS)

Figure S1 Contributions of null mutations to fitness by functional category. For each of 144 conditions, the fraction of members from each functional class with significantly beneficial (left) and deleterious (right) null mutations is shown. Values are normalized so that each column (condition) sums to 1. Values above each column show the total number of significant null mutations for the condition. A value of −1 indicates no significant mutations were found. Rows and columns were ordered by hierarchical clustering. Blue dots and red dots indicate data gathered in the BW25113 and MG1655 backgrounds, respectively. Gene categories are from the GenProtEC database.
(PDF)

Figure S2 Beneficial deletions that confer similar fitness increases cause distinct transcriptional changes. (A–B) Using the significance cutoff of an average fold-change of 2 (2 repetitions), expression of the indicated numbers of genes changed in the mutants compared to the parental strain in (A) alanine and (B) glutamine media. The corresponding estimated false discovery rates for alanine media are 17.4% (Δ*dnaJ*), 28.3% (Δ*gcvT*), 4.7% (Δ*cpxA*), and 7.5% (Δ*pgi*) and for glutamine media are 10.5%(Δ*dnaJ*), 10.0% (Δ*lrp*), 3.6% (Δ*hfq*), and 3.5% (Δ*cysQ*). See Materials and Methods for details. (C–D) Shown are functional categories identified by iPAGE [24] as enriched among the genes with decreased expression in (C) alanine or (D) glutamine media. (E–F) Shown are functional enrichments among the genes expressed at higher levels in the mutants in (E) alanine and (F) glutamine media. No significant functional depletions were identified.
(PDF)

Figure S3 Expression of chemotaxis and flagella biosynthesis genes. Shown is the expression of chemotaxis and flagella biosynthesis genes in mutants compared to the expression in the parental strain. Exponential phase cultures were grown in glutamine or alanine media as indicated.
(PDF)

Figure S4 Growth rates for deletion mutants in additional conditions. Growth rates for the strains of Figure 5 in defined media with (A) alanine, (B) glucose, (C) glutamine, or (D) asparagine as the sole carbon source (see Materials and Methods). Only data for conditions other than the one(s) in which the deletion was initially identified as advantageous are shown. Xs denote individual measurements. Red squares (black circles) denote mean growth rates for strains whose doubling time is (not) significantly different from that of the parental wild-type (WT) strain (2-sided Mann-Whitney test, 5% FDR calculated separately for the strains shown in each panel). Zero indicates no consistent growth, and these strains did not impact the FDR calculations. All tests in asparagine media lacked sufficient power for a finding of significance. Strains showing significant growth differences in glutamine had a q-value of 0.043. Significant q-values in glucose are as follows: *cpxA*: 0.00089, *crr*: 0.00089, *hfq*: 0.00089, *pgi*: 0.00089, *lrp*: 0.00201, *kgtP*: 0.00415, *cysQ*: 0.00460, *cpdA*: 0.01955, *pgm*: 0.04701.
(PDF)

Table S1 Literature examples of beneficial null mutations. ^a^ A deletion acts directly if the gene's product is at least as close to the key, fitness-relevant reaction as any other gene of the same functional category. ^b^ For some studies that identified multiple null mutations, only the best-characterized examples are included.
(DOC)

Table S2 Propensity of genes from different functional classes to generate beneficial or deleterious null mutations in the test conditions. ^a^ GenProtEC classifications (http://genprotec.mbl.edu/files/geneproductfunctions.txt) ^b^ The number of genes, excluding essential genes, included in each classification. Not all data sets report on each gene in each condition. ^c^ q-values for the enrichment of null mutations in a given class (maximum FDR at which the class would be deemed significant). Values significant at a 1% FDR are shown in bold.
(DOC)

Table S3 Null mutations beneficial in at least ten of the 144 conditions from our meta-analysis. ^a^ GenProtEC classifications (http://genprotec.mbl.edu/files/geneproductfunctions.txt).
(DOC)

Table S4 Strains used in this work. ^a^ Note that additional tests indicated that ZD42 (Δb3609) and ZD1 (Δb0015) also have an advantage over the parental strain in glutamine media, but these strains were not included in the preliminary testing in glutamine media; the fitness effect was found when testing the strains with an advantage in alanine media in the other media. We tested strains ZD14, ZD35, ZD38, and ZD57 in asparagine media because a preliminary analysis of the transposon insertion data suggested they might have a fitness advantage; none of the four strains grew faster than the parental strain, and in the final analysis of the transposon data none of the genes met the significance criteria. ^b^ Strains constructed for testing in alanine medium. ^c^ Strains constructed for testing in glutamine medium. ^d^ Strains constructed for testing in asparagine medium.
(DOC)

Table S5 Q-values for Figure 5. ^a^ We used a Mann-Whitney test (1-sided) to compare the doubling time of each mutant with the parental strain grown in the same media. See Table S4 for a complete list of the strains tested in each media. Considering all 48 growth tests simultaneously, we chose the significance cutoff to yield a false discovery rate less than 5% (q-value<0.05). False positive calculations include mutants tested in only the first stage of the asparagine or glutamine investigation.
(DOC)

Table S6 Significance of overlaps between sets of differentially expressed genes. Shown are the sizes of the overlaps among the sets of genes that increased or decreased at least 2-fold on average between the mutant and the parental strain in the indicated media. The probability of an overlap of the given size or larger occurring by chance was calculated using the hypergeometric distribution. P-values were adjusted for the 24 comparisons using a Bonferroni correction. Values greater than 0.05 are not shown.
(DOC)
