# Supplementary figures and images for: Bacterial Adaptation through Loss of Function
Source: PLoS Genet. 2013 Jul 11;9(7):e1003617. doi: 10.1371/journal.pgen.1003617 (PMC3708842; doi:10.1371/journal.pgen.1003617)

Gene class

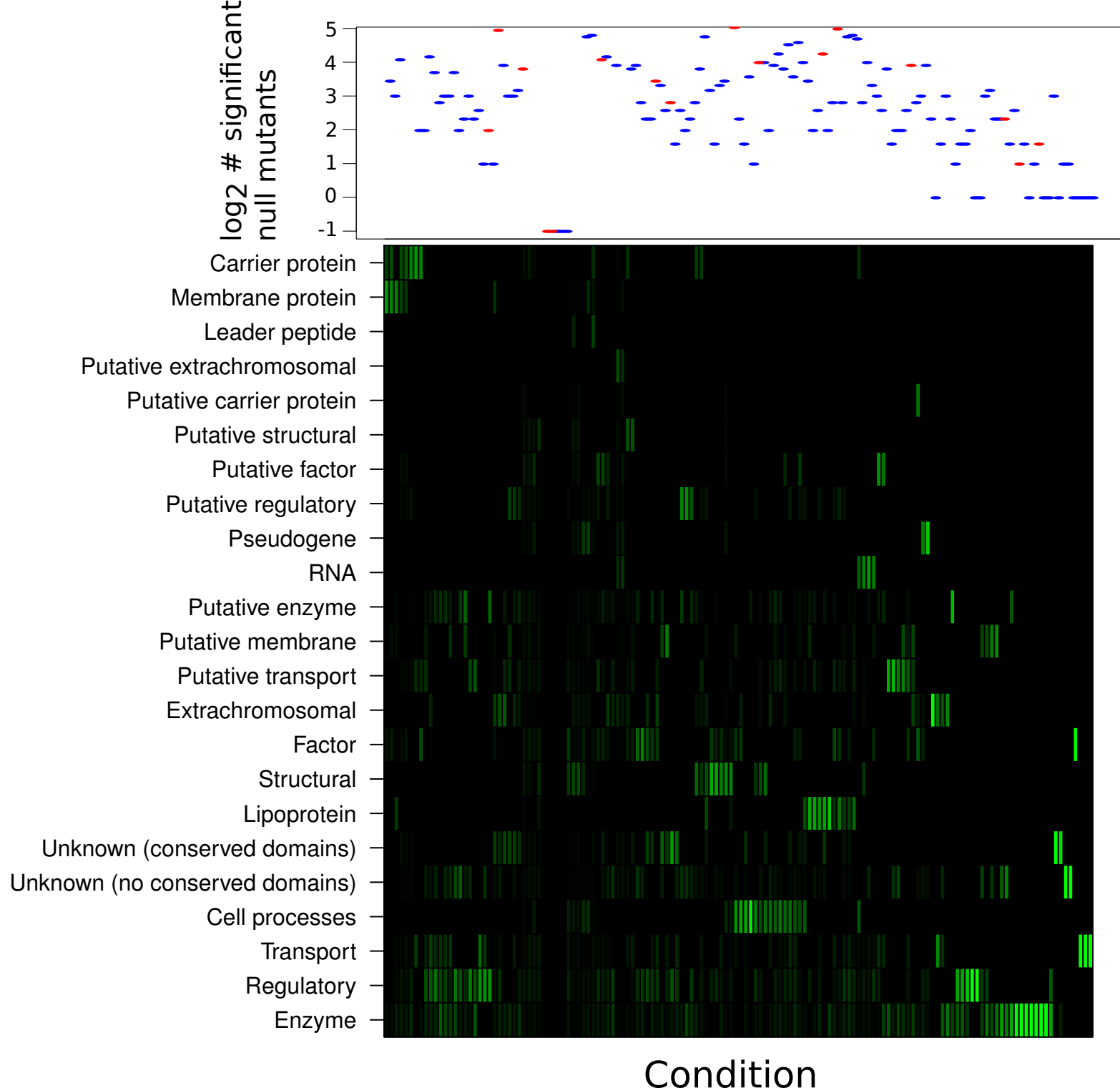

Gene class

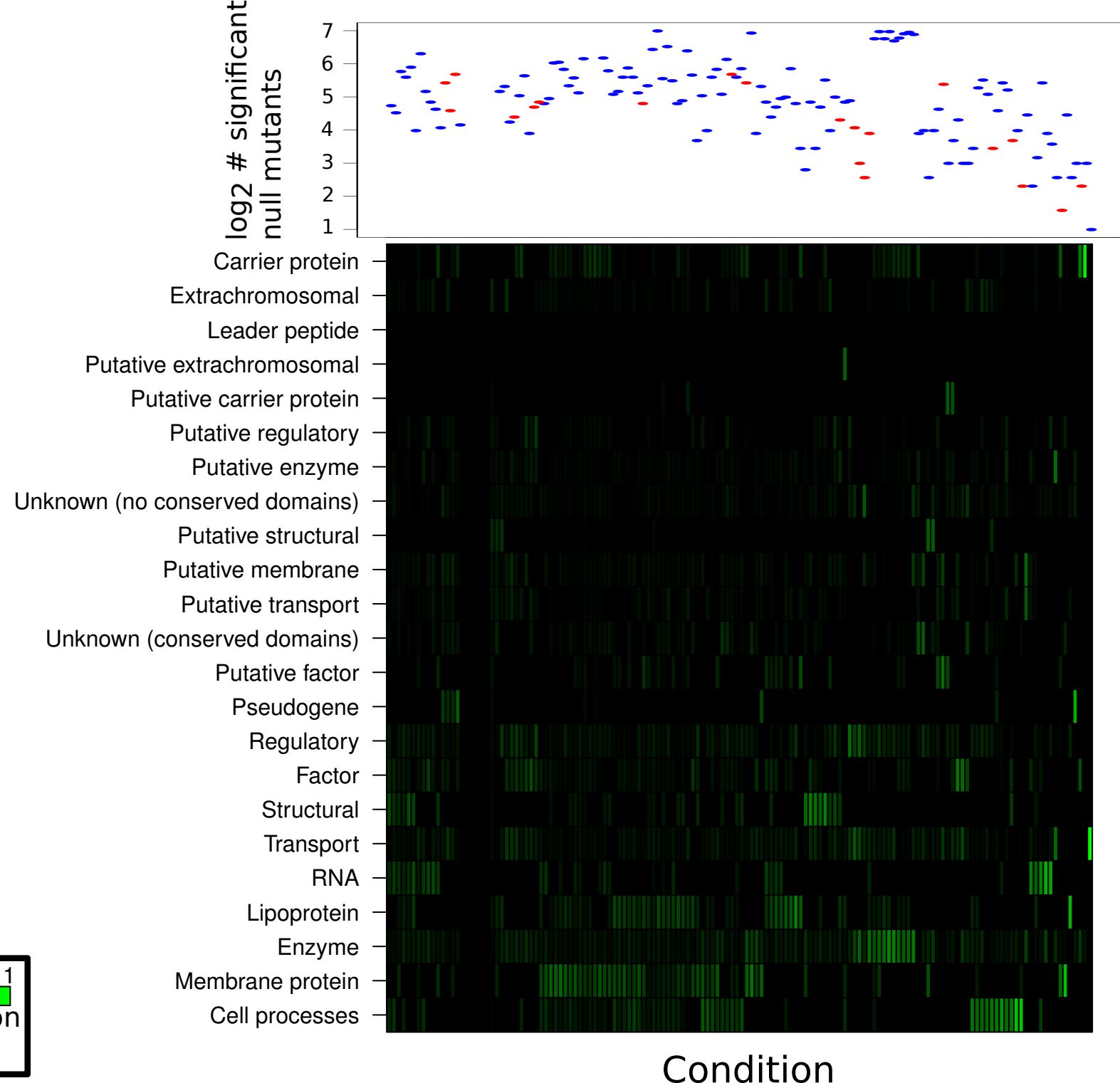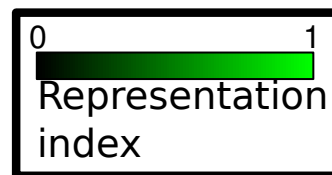

Supplement: Figure S1 — Contributions of null mutations to fitness by functional category. For each of 144 conditions, the fraction of members from each functional class with significantly beneficial (left) and deleterious (right) null mutations is shown. Values are normalized so that each column (condition) sums to 1. Values above each column show the total number of significant null mutations for the condition. A value of −1 indicates no significant mutations were found. Rows and columns were ordered by hierarchical clustering. Blue dots and red dots indicate data gathered in the BW25113 and MG1655 backgrounds, respectively. Gene categories are from the GenProtEC database. (PDF) [file pgen.1003617.s003.pdf]

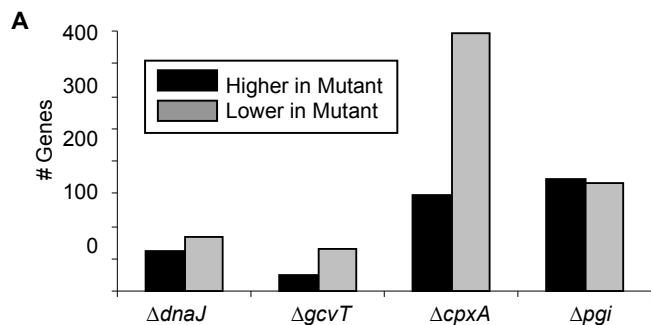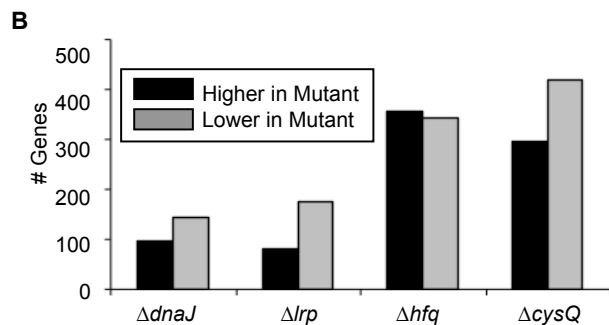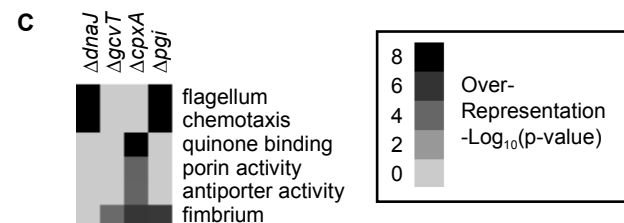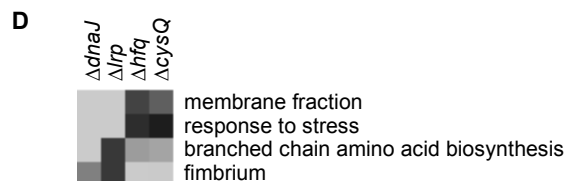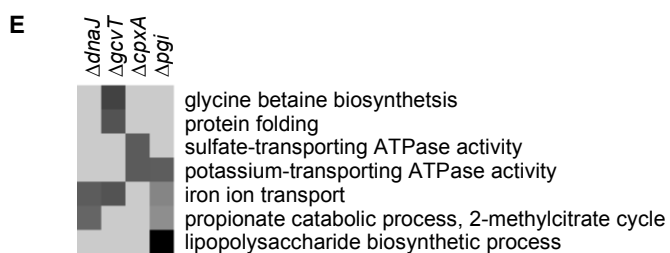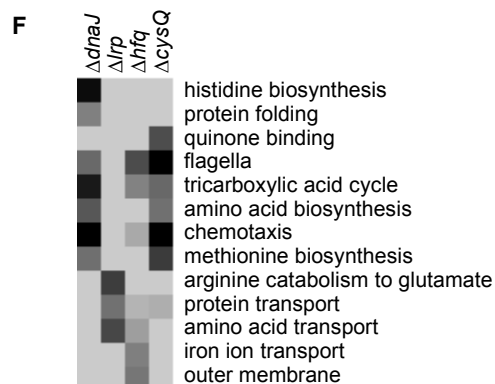

Supplement: Figure S2 — Beneficial deletions that confer similar fitness increases cause distinct transcriptional changes. (A–B) Using the significance cutoff of an average fold-change of 2 (2 repetitions), expression of the indicated numbers of genes changed in the mutants compared to the parental strain in (A) alanine and (B) glutamine media. The corresponding estimated false discovery rates for alanine media are 17.4% (ΔdnaJ), 28.3% (ΔgcvT), 4.7% (ΔcpxA), and 7.5% (Δpgi) and for glutamine media are 10.5%(ΔdnaJ), 10.0% (Δlrp), 3.6% (Δhfq), and 3.5% (ΔcysQ). See Materials and Methods for details. (C–D) Shown are functional categories identified by iPAGE [24] as enriched among the genes with decreased expression in (C) alanine or (D) glutamine media. (E–F) Shown are functional enrichments among the genes expressed at higher levels in the mutants in (E) alanine and (F) glutamine media. No significant functional depletions were identified. (PDF) [file pgen.1003617.s004.pdf]

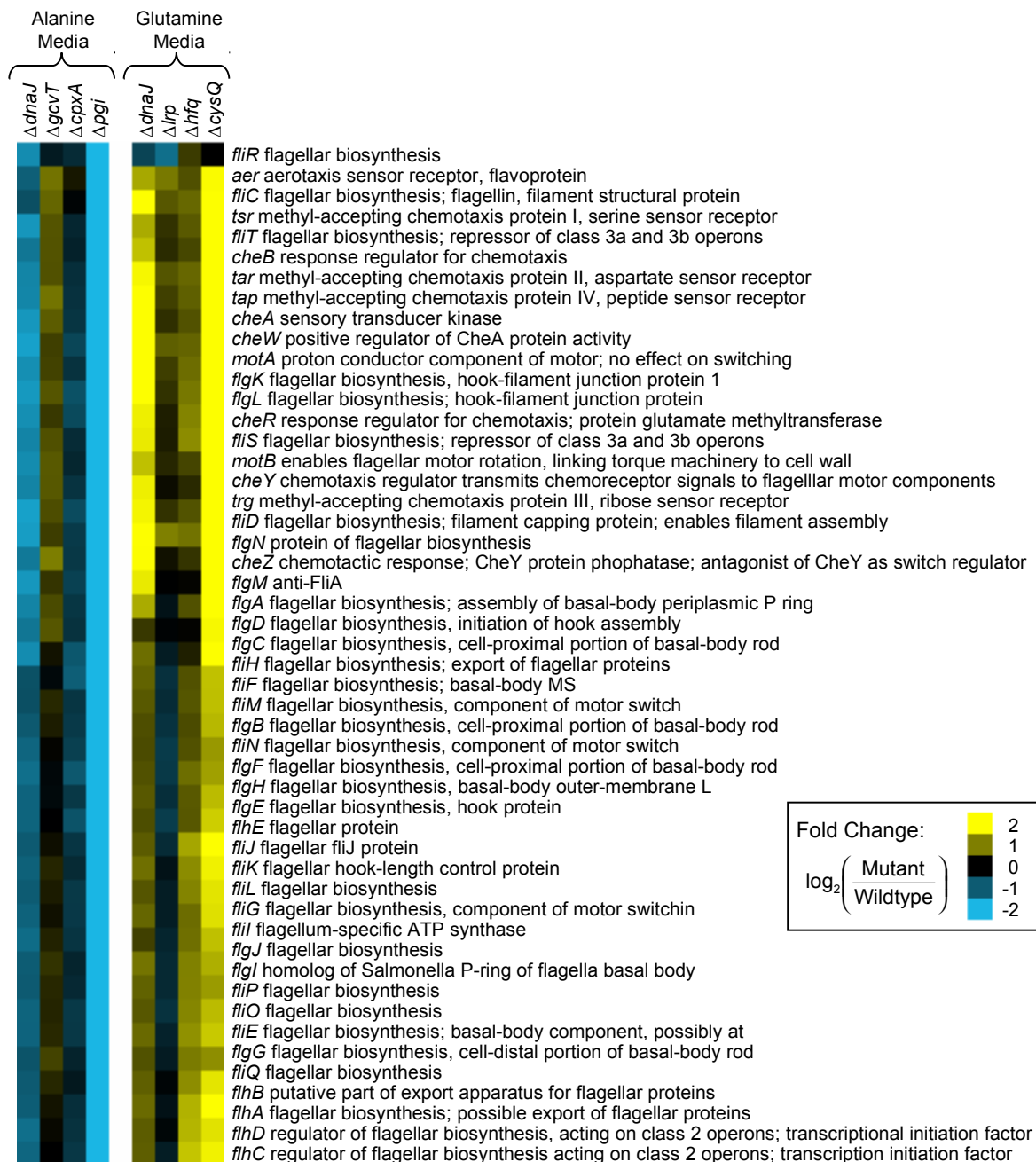

Supplement: Figure S3 — Expression of chemotaxis and flagella biosynthesis genes. Shown is the expression of chemotaxis and flagella biosynthesis genes in mutants compared to the expression in the parental strain. Exponential phase cultures were grown in glutamine or alanine media as indicated. (PDF) [file pgen.1003617.s005.pdf]

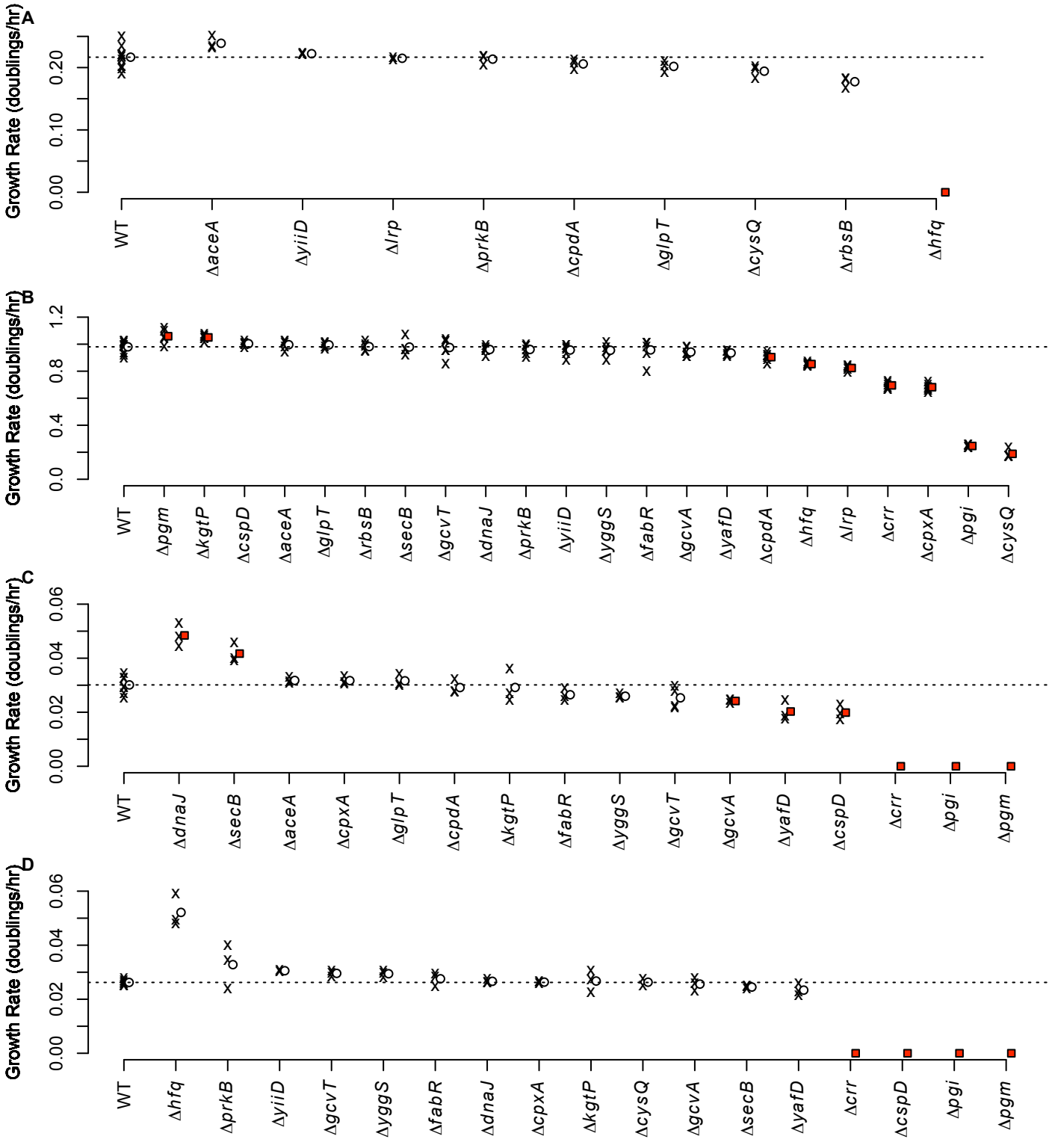

Supplement: Figure S4 — Growth rates for deletion mutants in additional conditions. Growth rates for the strains of Figure 5 in defined media with (A) alanine, (B) glucose, (C) glutamine, or (D) asparagine as the sole carbon source (see Materials and Methods). Only data for conditions other than the one(s) in which the deletion was initially identified as advantageous are shown. Xs denote individual measurements. Red squares (black circles) denote mean growth rates for strains whose doubling time is (not) significantly different from that of the parental wild-type (WT) strain (2-sided Mann-Whitney test, 5% FDR calculated separately for the strains shown in each panel). Zero indicates no consistent growth, and these strains did not impact the FDR calculations. All tests in asparagine media lacked sufficient power for a finding of significance. Strains showing significant growth differences in glutamine had a q-value of 0.043. Significant q-values in glucose are as follows: cpxA: 0.00089, crr: 0.00089, hfq: 0.00089, pgi: 0.00089, lrp: 0.00201, kgtP: 0.00415, cysQ: 0.00460, cpdA: 0.01955, pgm: 0.04701. (PDF) [file pgen.1003617.s006.pdf]
