# Supplementary material for: Bacterial Adaptation through Loss of Function
Source: PLoS Genet. 2013 Jul 11;9(7):e1003617. doi: 10.1371/journal.pgen.1003617 (PMC3708842; doi:10.1371/journal.pgen.1003617)
Supplement: Table S1 — Literature examples of beneficial null mutations. a A deletion acts directly if the gene's product is at least as close to the key, fitness-relevant reaction as any other gene of the same functional category. b For some studies that identified multiple null mutations, only the best-characterized examples are included. (DOC) [file pgen.1003617.s007.doc]

| Organism | Gene | Fitness Increase | Distance ^a^ | | | Functional Category | | | | | | Ref.^b^ |
| --- | --- | --- | --- | --- | --- | --- | --- | --- | --- | --- | --- | --- |
|  |  |  | Direct | Indirect | Unknown | Structural | Transporter | Enzymatic | Regulatory | Housekeeping | Unknown/Other |  |
| *Acinetobacter baylyi* | *ACIAD3309, ACIAD3383 (acr1)* | Increased triacylglycerol production |  | X |  |  |  | X |  |  |  |  |
| *Bacillus anthracis* | *plcR* | Increased sporulation frequency in the presence of the pXO1 plasmid . Reduced macrophage toxicity, which increases transport of the strain to peripheral lymph nodes . |  | X |  |  |  |  | X |  |  |  |
| *Burkholderia pseudomallei* | BPSS1219 | Ceftazidime resistance | X |  |  |  |  | X |  |  |  |  |
| *Cronobacter sakazakii* ES5 | *crtE*, *crtX*, *crtY* | Growth under osmotic stress |  |  | X |  |  | X |  |  |  |  |
| *C. sakazakii* ES5 | *crtY* | Resistance to oxidative stress | X |  |  |  |  | X |  |  |  |  |
| *C. sakazakii* ES5 | *crtE* | Resistance to tolyfluanid and aminoglycosides |  |  | X |  |  | X |  |  |  |  |
| *Escherichia coli* | *luxS* | Swimming motility |  | X |  |  |  | X |  |  |  |  |
| *E. coli* | *cls* | Freeze-thaw cycles | X |  |  |  |  | X |  |  |  |  |
| *E. coli* | *cspC* | Growth in broth at 37°C |  | X |  |  |  |  |  | X |  |  |
| *E. coli* | *zwf, frdA, ldhA, sfcA, maeB,* and *ndh* | Biomass yield on glucose |  | X |  |  |  | X |  |  |  |  |
| *E. coli* | ompR, envZ, | Motility in high salt |  | X |  |  |  |  | X |  |  |  |
| *E. coli* | znuA, znuC, yfiM | Motility in high salt |  |  | X |  |  |  |  |  | X |  |
| *E. coli* | Genes encoding flagella components | Bacteriophage χ resistance | X |  |  | X |  |  |  |  |  |  |
| *E. coli* | *mdoG*, *mdoH*, *rfaC*, *rfaI*, and other LPS biosynthesis genes | Bacteriophage χ resistance |  | X |  |  |  | X |  |  |  |  |
| *E. coli* | *acnB, gpsA, aceE, aceF, epd, gpmA, fbaA, lipA, lipB, pgk, pta, sdhB, tktA, tpiA, rpe, ubiX, ubiG, ubiF, ubiD, ispA, nfuA (gntY), cysB, serA, serB, cysE, feoC, iscR, nuoL, nuoK, nuoJ, nuoI, nuoH, nuoG, nuoF, nuoE, nuoC, nuoB, fre, ribD,* *ndh,*  *cyoE, hemC, cyoC, cyoB* | Aminoglycoside tolerance |  | X |  |  |  | X |  |  |  |  |
| *E. coli* | *crr, ptsI* | Aminoglycoside tolerance |  | X |  |  | X |  |  |  |  |  |
| *E. coli* | *fadR, pdhR* | Aminoglycoside tolerance |  | X |  |  |  |  | X |  |  |  |
| *E. coli* | *folX, folM* | Trimethoprim and sulfamonomethoxine tolerance |  | X |  |  |  | X |  |  |  |  |
| *E. coli* | *mdoG, mdoH* | Beta-lactam tolerance |  | X |  |  |  | X |  |  |  |  |
| *E. coli* | *rcsD*, *rcsC* | Beta-lactam tolerance |  | X |  |  |  |  | X |  |  |  |
| *E. coli* | *slt* | Increased ethanol tolerance | X |  |  |  |  | X |  |  |  |  |
| *E. coli* | *fnr*, *arcA* | Increased ethanol tolerance | X |  |  |  |  |  | X |  |  |  |
| *E. coli* | *cafA* | Increased ethanol tolerance | X |  |  |  |  |  |  | X |  |  |
| *E. coli* | fimbrial genes | Increased ethanol tolerance | X |  |  | X |  |  |  |  |  |  |
| *E. coli* | *rbs* operon | Glucose media |  | X |  |  |  | X |  |  |  |  |
| *E. coli* | *glpR* | Media with glycerol | X |  |  |  |  |  | X |  |  |  |
| *E. coli* | *lrp* | Stationary phase |  | X |  |  |  |  | X |  |  |  |
| *E. coli* | *fliM, fliR, flgE* | LB media | X |  |  | X |  |  |  |  |  |  |
| *E. coli* | *kil* | Bicyclomycin tolerance | X |  |  |  |  |  |  |  |  |  |
| *E. coli* | *hfq* | Bicyclomycin tolerance |  | X |  |  |  |  |  | X |  |  |
| *E. coli* | *hda* | Ciprofloxacin tolerance |  | X |  |  |  |  |  | X |  |  |
| *E. coli* | *gshA, mnmA, feaB, trpB, yncB, iscS* | Ciprofloxacin tolerance |  | X |  |  |  | X |  |  |  |  |
| *E. coli* | *flgA* | Ciprofloxacin tolerance |  |  | X |  |  |  |  |  | X |  |
| *E. coli* | *fur, b1506* | Ciprofloxacin tolerance |  |  | X |  |  |  | X |  |  |  |
| *E. coli* | *lrp* | Asparagine as sole carbon source |  |  | X |  |  |  | X |  |  |  |
| *E. coli* | *rpoS* | Glucose or nitrogen-limited chemostats |  | X |  |  |  |  | X |  |  |  |
| *E. coli* | *crl* | Stationary phase |  | X |  |  |  |  | X |  |  |  |
| *E. coli* | *ompF* | Tetracycline resistance | X |  |  |  | X |  |  |  |  |  |
| *E. coli* | *cysH, icdA, metE, purB* | Nalidixic acid tolerance |  | X |  |  |  | X |  |  |  |  |
| *Klebsiella pneumoniae* | KP1_0420  *(sugE)* | Biofilm formation |  |  | X |  |  |  |  |  | X |  |
| *K. pneumoniae* | *ompK36* | Cefoxitin tolerance | X |  |  |  | X |  |  |  |  |  |
| *Mycobacterium smegmatis* | *MSMEG0392 (gtf2)* | Phage I3 resistance | X |  |  |  |  | X |  |  |  |  |
| *M. smegmatis* and *M. tuberculosis* | *Rv1694 (tlyA)* | Capreomycin tolerance | X |  |  |  |  | X |  |  |  |  |
| *M. tuberculosis* | *fadD23* | Increased macrophage binding | X |  |  |  |  | X |  |  |  |  |
| *Mycoplasma genitalium* | *MG414, MG415* | SP4 media |  |  | X |  |  |  |  |  | X |  |
| *Mycoplasma genitalium* | *MG460* | SP4 media |  |  | X |  |  | X |  |  |  |  |
| *Porphyromonas gingivalis* | *KDP400 (gtfB)* | Biofilm formation | X |  |  |  |  | X |  |  |  |  |
| *Pseudomonas aeruginosa* | *hmgA* | Oxidative stress resistance, persistence in chronic lung infection | X |  |  |  |  | X |  |  |  |  |
| *P. aeruginosa* | PA0614, *nuoA*, *nuoK*, PA2653, PA2771, PA3048, PA3222, *rhlI*, PA3844, PA5207, *wzm* | Tobramycin tolerance in biofilms |  | X |  |  |  | X |  |  |  |  |
| *P. aeruginosa* | *lasR* | Tobramycin tolerance in biofilms |  | X |  |  |  |  | X |  |  |  |
| *P. aeruginosa* | PA1329, PA3726, PA3966 | Tobramycin tolerance in biofilms |  |  | X |  |  |  |  |  | X |  |
| *P. aeruginosa* | *nuoA*, *nuoK*, PA2653, PA2771, PA3048, PA3222, *rhlI*, PA3844, PA5207, *wzm* | Tobramycin tolerance in planktonic cultures |  | X |  |  |  | X |  |  |  |  |
| *P. aeruginosa* | *lasR* | Tobramycin tolerance in planktonic cultures |  | X |  |  |  |  | X |  |  |  |
| *P. aeruginosa* | PA1329, PA3966 | Tobramycin tolerance in planktonic cultures |  |  | X |  |  |  |  |  | X |  |
| *P. aeruginosa* | *PA1856, PA2998 (nqrB), PA3478 (rlhB),* | Chronic airway infection |  | X |  |  |  | X |  |  |  |  |
| *P. aeruginosa* | *PA0890 (aotM), PA2252, PA4887* | Chronic airway infection |  | X |  |  | X |  |  |  |  |  |
| *P. aeruginosa* | *PA4554 (pilY1), PA0410 (pilI), PA1077 (flgB)* | Chronic airway infection |  | X |  | X |  |  |  |  |  |  |
| *P. aeruginosa* | *PA5053 (hslV), PA0499* | Chronic airway infection |  | X |  |  |  |  |  |  |  |  |
| *P. aeruginosa* | PA2972, PA5028 | Chronic airway infection |  |  | X |  |  |  |  |  | X |  |
| *P. aeruginosa* | *lasR* | Ceftazidime tolerance; growth with phenylalanine, isoleucine, and tyrosine |  | X |  |  |  |  | X |  |  |  |
| *P. aeruginosa* | *pilY1* | Resistance to killing by neutrophils, growth in stationary phase, survival in murine airway infection models | X |  |  | X |  |  |  |  |  |  |
| *P. aeruginosa* | *mucA* | Protection from reactive oxygen intermediates, resistance to host immune response |  | X |  |  |  |  | X |  |  |  |
| *P. aeruginosa* | *PA2491 (mexS)* | Tolerance to chloramphenicol, quinolones, and imipenem |  | X |  |  |  | X |  |  |  |  |
| *P. aeruginosa* | *PA3574 (nalD)* | Tolerance to chloramphenicol, quinolones, and imipenem |  |  | X |  |  |  | X |  |  |  |
| *P. aeruginosa* | *mexR* | Ticarcillin and aztreonam resistance | X |  |  |  |  |  | X |  |  |  |
| *P. aeruginosa* | *PA3721 (nalC)* | Multi-drug resistance |  | X |  |  |  |  | X |  |  |  |
| *P. aeruginosa* | *wapR*, PA5001, PA5002, PA5003,  PA5005, *wpmM, wbpL, wspE, galU* | Ceftazidime, imipenem, or meropenem tolerance | X |  |  |  |  | X |  |  |  |  |
| *P. aeruginosa* | *ampR* | Ceftazidime, imipenem, or meropenem tolerance | X |  |  |  |  |  | X |  |  |  |
| *P. aeruginosa* | *ampD*, *mpl*, *dacB* | Ceftazidime, imipenem, or meropenem tolerance |  | X |  |  |  | X |  |  |  |  |
| *P. aeruginosa* | *mexZ* | Aminoglycoside resistance | X |  |  |  |  |  | X |  |  |  |
| *P. aeruginosa* | *cspD* | Increased swarming |  | X |  |  |  |  | X |  |  |  |
| *P. aeruginosa* | Various | Tobramycin resistance | X | X | X |  |  | X | X |  | X |  |
| *P. aeruginosa* | Various | Resistance to ciprofloxacin | X | X | X |  |  | X | X | X | X |  |
| *P. aeruginosa* | *mexT* | *nfxC*-type antibiotic tolerance | X |  |  |  |  |  | X |  |  |  |
| *Pseudomonas putida* | *dsbA* | Biofilm formation |  |  | X |  |  | X |  |  |  |  |
| *Salmonella enterica* | *oxyS* | Colonization of spleen after intraperitoneal infection |  | X |  |  |  |  | X |  |  |  |
| *Shigella flexneri* | *cadA* | Increased fluid secretion in host cells . Increased infiltration of polymorphonuclear leucocytes across intestinal epithelium in host . | X |  |  |  |  | X |  |  |  |  |
| *S. flexneri* | *nadA*, *nadB* | Virulence, invasion of host cells | X |  |  |  |  | X |  |  |  |  |
| *S. flexneri* | *speG* | Survival under oxidative stress | X |  |  |  |  | X |  |  |  |  |
| *Staphylococcus aureus* | *mecA* | Growth with vancomycin (deletion improves growth of vancomycin-resistant strains) | X |  |  |  |  | X |  |  |  |  |
| *S. aureus* | *codY* | Increased biofilm robustness |  |  | X |  |  |  | X |  |  |  |
| *Staphylococcus epidermidis* | *icaADBC* | Colonization of human skin | X |  |  |  |  | X |  |  |  |  |
| *Streptococcus pneumoniae* | *ciaRH* | Increased competence |  |  | X |  |  |  | X |  |  |  |
| *S. pneumoniae* | *comE* | Improved colonization of an infant rat model |  |  | X |  |  |  | X |  |  |  |
| *S. pneumoniae* | *SP_1697, SP_1464, SP_1463, SP_1421, SP_0593* | Exponential growth on Todd Hewitt broth supplemented with  yeast extract and Oxyrase |  |  | X |  |  |  |  |  | X |  |

**References**

1. Santala S, Efimova E, Kivinen V, Larjo A, Aho T, et al. (2011) Improved triacylglycerol production in *Acinetobacter baylyi* ADP1 by metabolic engineering. Microb Cell Fact 10: 36.

2. Mignot T, Mock M, Robichon D, Landier A, Lereclus D, et al. (2001) The incompatibility between the PlcR- and AtxA-controlled regulons may have selected a nonsense mutation in *Bacillus anthracis*. Mol Microbiol 42: 1189-1198.

3. Sastalla I, Maltese LM, Pomerantseva OM, Pomerantsev AP, Keane-Myers A, et al. (2010) Activation of the latent PlcR regulon in *Bacillus anthracis*. Microbiology 156: 2982-2993.

4. Chantratita N, Rholl DA, Sim B, Wuthiekanun V, Limmathurotsakul D, et al. (2011) Antimicrobial resistance to ceftazidime involving loss of penicillin-binding protein 3 in *Burkholderia pseudomallei*. Proc Natl Acad Sci U S A 108: 17165-17170.

5. Johler S, Stephan R, Hartmann I, Kuehner KA, Lehner A (2010) Genes involved in yellow pigmentation of *Cronobacter sakazakii* ES5 and influence of pigmentation on persistence and growth under environmental stress. Appl Environ Microbiol 76: 1053-1061.

6. Ling H, Kang A, Tan MH, Qi X, Chang MW (2010) The absence of the *luxS* gene increases swimming motility and flagella synthesis in *Escherichia coli* K12. Biochem Biophys Res Commun 401: 521-526.

7. Sleight SC, Orlic C, Schneider D, Lenski RE (2008) Genetic basis of evolutionary adaptation by *Escherichia coli* to stressful cycles of freezing, thawing and growth. Genetics 180: 431-443.

8. Rath D, Jawali N (2006) Loss of expression of *cspC*, a cold shock family gene, confers a gain of fitness in *Escherichia coli* K-12 strains. J Bacteriol 188: 6780-6785.

9. Trinh CT, Carlson R, Wlaschin A, Srienc F (2006) Design, construction and performance of the most efficient biomass producing *E. coli* bacterium. Metab Eng 8: 628-638.

10. Girgis HS, Liu Y, Ryu WS, Tavazoie S (2007) A comprehensive genetic characterization of bacterial motility. PLoS Genet 3: 1644-1660.

11. Girgis HS, Hottes AK, Tavazoie S (2009) Genetic architecture of intrinsic antibiotic susceptibility. PLoS One 4: e5629.

12. Goodarzi H, Bennett BD, Amini S, Reaves ML, Hottes AK, et al. (2010) Regulatory and metabolic rewiring during laboratory evolution of ethanol tolerance in *E. coli*. Mol Syst Biol 6: 378.

13. Cooper VS, Schneider D, Blot M, Lenski RE (2001) Mechanisms causing rapid and parallel losses of ribose catabolism in evolving populations of *Escherichia coli* B. J Bacteriol 183: 2834-2841.

14. Freddolino PL, Amini S, Tavazoie S (2012) Newly identified genetic variations in common *Escherichia coli* MG1655 stock cultures. J Bacteriol 194: 303-306.

15. Zinser ER, Kolter R (2000) Prolonged stationary-phase incubation selects for *lrp* mutations in *Escherichia coli* K-12. J Bacteriol 182: 4361-4365.

16. Edwards RJ, Sockett RE, Brookfield JF (2002) A simple method for genome-wide screening for advantageous insertions of mobile DNAs in *Escherichia coli*. Curr Biol 12: 863-867.

17. Tran L, van Baarsel JA, Washburn RS, Gottesman ME, Miller JH (2011) Single-gene deletion mutants of *Escherichia coli* with altered sensitivity to bicyclomycin, an inhibitor of transcription termination factor Rho. J Bacteriol 193: 2229-2235.

18. Tamae C, Liu A, Kim K, Sitz D, Hong J, et al. (2008) Determination of antibiotic hypersensitivity among 4,000 single-gene-knockout mutants of *Escherichia coli*. J Bacteriol 190: 5981-5988.

19. Goodarzi H, Hottes AK, Tavazoie S (2009) Global discovery of adaptive mutations. Nat Methods 6: 581-583.

20. Notley-McRobb L, King T, Ferenci T (2002) rpoS mutations and loss of general stress resistance in *Escherichia coli* populations as a consequence of conflict between competing stress responses. J Bacteriol 184: 806-811.

21. Madan R, Moorthy S, Mahadevan S (2008) Enhanced expression of the *bgl* operon of *Escherichia coli* in the stationary phase. FEMS Microbiol Lett 288: 131-139.

22. Thanassi DG, Suh GS, Nikaido H (1995) Role of outer membrane barrier in efflux-mediated tetracycline resistance of *Escherichia coli*. J Bacteriol 177: 998-1007.

23. Helling RB, Janes BK, Kimball H, Tran T, Bundesmann M, et al. (2002) Toxic waste disposal in *Escherichia coli*. J Bacteriol 184: 3699-3703.

24. Wu MC, Lin TL, Hsieh PF, Yang HC, Wang JT (2011) Isolation of genes involved in biofilm formation of a *Klebsiella pneumoniae* strain causing pyogenic liver abscess. PLoS One 6: e23500.

25. Hernandez-Alles S, Benedi VJ, Martinez-Martinez L, Pascual A, Aguilar A, et al. (1999) Development of resistance during antimicrobial therapy caused by insertion sequence interruption of porin genes. Antimicrob Agents Chemother 43: 937-939.

26. Chen J, Kriakov J, Singh A, Jacobs WR, Jr., Besra GS, et al. (2009) Defects in glycopeptidolipid biosynthesis confer phage I3 resistance in *Mycobacterium smegmatis*. Microbiology 155: 4050-4057.

27. Maus CE, Plikaytis BB, Shinnick TM (2005) Mutation of *tlyA* confers capreomycin resistance in *Mycobacterium tuberculosis*. Antimicrob Agents Chemother 49: 571-577.

28. Lynett J, Stokes RW (2007) Selection of transposon mutants of *Mycobacterium tuberculosis* with increased macrophage infectivity identifies *fadD23* to be involved in sulfolipid production and association with macrophages. Microbiology 153: 3133-3140.

29. Glass JI, Assad-Garcia N, Alperovich N, Yooseph S, Lewis MR, et al. (2006) Essential genes of a minimal bacterium. Proc Natl Acad Sci U S A 103: 425-430.

30. Yamaguchi M, Sato K, Yukitake H, Noiri Y, Ebisu S, et al. (2010) A *Porphyromonas gingivalis* mutant defective in a putative glycosyltransferase exhibits defective biosynthesis of the polysaccharide portions of lipopolysaccharide, decreased gingipain activities, strong autoaggregation, and increased biofilm formation. Infect Immun 78: 3801-3812.

31. Rodriguez-Rojas A, Mena A, Martin S, Borrell N, Oliver A, et al. (2009) Inactivation of the *hmgA* gene of *Pseudomonas aeruginosa* leads to pyomelanin hyperproduction, stress resistance and increased persistence in chronic lung infection. Microbiology 155: 1050-1057.

32. Amini S, Hottes AK, Smith LE, Tavazoie S (2011) Fitness Landscape of Antibiotic Tolerance in *Pseudomonas aeruginosa* Biofilms. PLoS Pathog 7: e1002298.

33. Bianconi I, Milani A, Cigana C, Paroni M, Levesque RC, et al. (2011) Positive signature-tagged mutagenesis in *Pseudomonas aeruginosa*: tracking patho-adaptive mutations promoting airways chronic infection. PLoS Pathog 7: e1001270.

34. D'Argenio DA, Wu M, Hoffman LR, Kulasekara HD, Deziel E, et al. (2007) Growth phenotypes of *Pseudomonas aeruginosa* *lasR* mutants adapted to the airways of cystic fibrosis patients. Mol Microbiol 64: 512-533.

35. Bohn YS, Brandes G, Rakhimova E, Horatzek S, Salunkhe P, et al. (2009) Multiple roles of *Pseudomonas aeruginosa* TBCF10839 PilY1 in motility, transport and infection. Mol Microbiol 71: 730-747.

36. Moyano AJ, Lujan AM, Argarana CE, Smania AM (2007) MutS deficiency and activity of the error-prone DNA polymerase IV are crucial for determining *mucA* as the main target for mucoid conversion in *Pseudomonas aeruginosa*. Mol Microbiol 64: 547-559.

37. Sobel ML, Neshat S, Poole K (2005) Mutations in PA2491 (*mexS*) promote MexT-dependent *mexEF*-*oprN* expression and multidrug resistance in a clinical strain of *Pseudomonas aeruginosa*. J Bacteriol 187: 1246-1253.

38. Sobel ML, Hocquet D, Cao L, Plesiat P, Poole K (2005) Mutations in PA3574 (*nalD*) lead to increased MexAB-OprM expression and multidrug resistance in laboratory and clinical isolates of *Pseudomonas aeruginosa*. Antimicrob Agents Chemother 49: 1782-1786.

39. Boutoille D, Corvec S, Caroff N, Giraudeau C, Espaze E, et al. (2004) Detection of an IS21 insertion sequence in the *mexR* gene of *Pseudomonas aeruginosa* increasing beta-lactam resistance. FEMS Microbiol Lett 230: 143-146.

40. Cao L, Srikumar R, Poole K (2004) MexAB-OprM hyperexpression in NalC-type multidrug-resistant *Pseudomonas aeruginosa*: identification and characterization of the *nalC* gene encoding a repressor of PA3720-PA3719. Mol Microbiol 53: 1423-1436.

41. Alvarez-Ortega C, Wiegand I, Olivares J, Hancock RE, Martinez JL (2010) Genetic determinants involved in the susceptibility of *Pseudomonas aeruginosa* to beta-lactam antibiotics. Antimicrob Agents Chemother 54: 4159-4167.

42. Llanes C, Hocquet D, Vogne C, Benali-Baitich D, Neuwirth C, et al. (2004) Clinical strains of *Pseudomonas aeruginosa* overproducing MexAB-OprM and MexXY efflux pumps simultaneously. Antimicrob Agents Chemother 48: 1797-1802.

43. Yeung AT, Torfs EC, Jamshidi F, Bains M, Wiegand I, et al. (2009) Swarming of *Pseudomonas aeruginosa* is controlled by a broad spectrum of transcriptional regulators, including MetR. J Bacteriol 191: 5592-5602.

44. Schurek KN, Marr AK, Taylor PK, Wiegand I, Semenec L, et al. (2008) Novel genetic determinants of low-level aminoglycoside resistance in *Pseudomonas aeruginosa*. Antimicrob Agents Chemother 52: 4213-4219.

45. Breidenstein EB, Khaira BK, Wiegand I, Overhage J, Hancock RE (2008) Complex ciprofloxacin resistome revealed by screening a *Pseudomonas aeruginosa* mutant library for altered susceptibility. Antimicrob Agents Chemother 52: 4486-4491.

46. Maseda H, Saito K, Nakajima A, Nakae T (2000) Variation of the *mexT* gene, a regulator of the MexEF-oprN efflux pump expression in wild-type strains of *Pseudomonas aeruginosa*. FEMS Microbiol Lett 192: 107-112.

47. Lee Y, Oh S, Park W (2009) Inactivation of the *Pseudomonas putida* KT2440 *dsbA* gene promotes extracellular matrix production and biofilm formation. FEMS Microbiol Lett 297: 38-48.

48. Santiviago CA, Reynolds MM, Porwollik S, Choi SH, Long F, et al. (2009) Analysis of pools of targeted *Salmonella* deletion mutants identifies novel genes affecting fitness during competitive infection in mice. PLoS Pathog 5: e1000477.

49. Maurelli AT, Fernández RE, Bloch CA, Rode CK, Fasano A (1998) “Black holes” and bacterial pathogenicity: A large genomic deletion that enhances the virulence of *Shigella* spp. and enteroinvasive *Escherichia coli*. Proc Natl Acad Sci U S A 95: 3943-3948.

50. McCormick BA, Fernandez MI, Siber AM, Maurelli AT (1999) Inhibition of *Shigella flexneri*-induced transepithelial migration of polymorphonuclear leucocytes by cadaverine. Cell Microbiol 1: 143-155.

51. Prunier A-L, Schuch R, Fernandez RE, Mumy KL, Kohler H, et al. (2007) *nadA* and *nadB* of *Shigella flexneri* 5a are antivirulence loci responsible for the synthesis of quinolinate, a small molecule inhibitor of *Shigella* pathogenicity. Microbiology 153: 2363-2372.

52. Barbagallo M, Di Martino ML, Marcocci L, Pietrangeli P, De Carolis E, et al. (2011) A New Piece of the *Shigella* Pathogenicity Puzzle: Spermidine Accumulation by Silencing of the *speG* Gene. PLoS One 6: e27226.

53. Noto MJ, Fox PM, Archer GL (2008) Spontaneous deletion of the methicillin resistance determinant, *mecA*, partially compensates for the fitness cost associated with high-level vancomycin resistance in *Staphylococcus aureus*. Antimicrob Agents Chemother 52: 1221-1229.

54. Majerczyk CD, Sadykov MR, Luong TT, Lee C, Somerville GA, et al. (2008) *Staphylococcus aureus* CodY negatively regulates virulence gene expression. J Bacteriol 190: 2257-2265.

55. Rogers KL, Rupp ME, Fey PD (2008) The presence of *icaADBC* is detrimental to the colonization of human skin by *Staphylococcus epidermidis*. Appl Environ Microbiol 74: 6155-6157.

56. Echenique JR, Chapuy-Regaud S, Trombe MC (2000) Competence regulation by oxygen in *Streptococcus pneumoniae*: involvement of *ciaRH* and *comCDE*. Mol Microbiol 36: 688-696.

57. Kowalko JE, Sebert ME (2008) The *Streptococcus pneumoniae* competence regulatory system influences respiratory tract colonization. Infect Immun 76: 3131-3140.

58. van Opijnen T, Bodi KL, Camilli A (2009) Tn-seq: high-throughput parallel sequencing for fitness and genetic interaction studies in microorganisms. Nat Methods 6: 767-772.
