# Supplementary material for: Bacterial Adaptation through Loss of Function
Source: PLoS Genet. 2013 Jul 11;9(7):e1003617. doi: 10.1371/journal.pgen.1003617 (PMC3708842; doi:10.1371/journal.pgen.1003617)
Supplement: Table S2 — Propensity of genes from different functional classes to generate beneficial or deleterious null mutations in the test conditions. a GenProtEC classifications (http://genprotec.mbl.edu/files/geneproductfunctions.txt) b The number of genes, excluding essential genes, included in each classification. Not all data sets report on each gene in each condition. c q-values for the enrichment of null mutations in a given class (maximum FDR at which the class would be deemed significant). Values significant at a 1% FDR are shown in bold. (DOC) [file pgen.1003617.s008.doc]

| **Category** ^a^ | **Genes**  **In**  **Class** ^b^ | **Enrichment of beneficial null mutations (q)** ^c^ | **Enrichment of detrimental null mutations (q)** ^c^ |
| --- | --- | --- | --- |
| Carrier protein | 69 | 0.159 | 0.514 |
| Cell processes | 37 | 0.022 | **<0.001** |
| Unknown (has conserved domains) | 136 | 1.000 | 1.000 |
| Enzyme | 969 | **<0.001** | **<0.001** |
| Factor | 120 | 0.159 | 1.000 |
| Extrachromosomal | 295 | 1.000 | 1.000 |
| Lipoprotein | 44 | 1.000 | **<0.001** |
| Membrane protein | 42 | 1.000 | **<0.001** |
| Regulatory | 250 | **<0.001** | **<0.001** |
| Structural | 44 | **<0.001** | 1.000 |
| Transport | 347 | 1.000 | 1.000 |
| Pseudogene | 64 | 1.000 | 1.000 |
| Putative carrier protein | 41 | 1.000 | 1.000 |
| Putative enzyme | 359 | 1.000 | 1.000 |
| Putative factor | 56 | 1.000 | 1.000 |
| Putative extrachromosomal | 11 | 1.000 | 1.000 |
| Putative membrane | 201 | 1.000 | 1.000 |
| Putative regulatory | 152 | 1.000 | 1.000 |
| Putative transport | 232 | 1.000 | 1.000 |
| Putative structural | 37 | 1.000 | 1.000 |
| RNA | 141 | **<0.001** | 1.000 |
| Leader peptide | 11 | 1.000 | 1.000 |
| Unknown (no conserved domains) | 455 | 1.000 | 1.000 |
