# Supplementary material for: Bacterial Adaptation through Loss of Function
Source: PLoS Genet. 2013 Jul 11;9(7):e1003617. doi: 10.1371/journal.pgen.1003617 (PMC3708842; doi:10.1371/journal.pgen.1003617)
Supplement: Table S3 — Null mutations beneficial in at least ten of the 144 conditions from our meta-analysis. a GenProtEC classifications (http://genprotec.mbl.edu/files/geneproductfunctions.txt). (DOC) [file pgen.1003617.s009.doc]

| Name | Category^a^ | Description | # beneficial conditions | # deleterious conditions |
| --- | --- | --- | --- | --- |
| *lon* | Enzyme | Housekeeping protease | 27 | 9 |
| *mdoH* | Enzyme | Periplasmic glucan synthesis | 14 | 2 |
| *waaP* | Enzyme | LPS biosynthetic protein | 14 | 41 |
| *fis* | Regulatory | Transcription factor | 13 | 23 |
| *dnaJ* | Factor | Heat shock chaperone | 12 | 20 |
| *mdoG* | Enzyme | Periplasmic glucan synthesis | 11 | 1 |
| *oxyR* | Regulatory | Transcription factor | 11 | 0 |

^a^ GenProtEC classifications (<http://genprotec.mbl.edu/files/geneproductfunctions.txt>)
