# Supplementary material for: Bacterial Adaptation through Loss of Function
Source: PLoS Genet. 2013 Jul 11;9(7):e1003617. doi: 10.1371/journal.pgen.1003617 (PMC3708842; doi:10.1371/journal.pgen.1003617)
Supplement: Table S4 — Strains used in this work. a Note that additional tests indicated that ZD42 (Δb3609) and ZD1 (Δb0015) also have an advantage over the parental strain in glutamine media, but these strains were not included in the preliminary testing in glutamine media; the fitness effect was found when testing the strains with an advantage in alanine media in the other media. We tested strains ZD14, ZD35, ZD38, and ZD57 in asparagine media because a preliminary analysis of the transposon insertion data suggested they might have a fitness advantage; none of the four strains grew faster than the parental strain, and in the final analysis of the transposon data none of the genes met the significance criteria. b Strains constructed for testing in alanine medium. c Strains constructed for testing in glutamine medium. d Strains constructed for testing in asparagine medium. (DOC) [file pgen.1003617.s010.doc]

| **Strain Number** | **Genotype** | **Source** |
| --- | --- | --- |
| ZD1^a,b^ | AH28 ∆b0015 | Transduced in allele from Keio collection and removed kan marker. |
| ZD2^c^ | AH28 ∆b0107 | Transduced in allele from Keio collection and removed kan marker. |
| ZD3^b^ | AH28 ∆b0209 | Transduced in allele from Keio collection and removed kan marker. |
| ZD5^b^ | AH28 ∆b0688 | Transduced in allele from Keio collection and removed kan marker. |
| ZD6^b^ | AH28 ∆b0880 | Transduced in allele from Keio collection and removed kan marker. |
| ZD7^d^ | AH28 ∆b0888 | Transduced in allele from Keio collection and removed kan marker. |
| ZD8^c,d^ | AH28 ∆b0889 | Transduced in allele from Keio collection and removed kan marker. |
| ZD10^b^ | AH28 ∆b1170 | Transduced in allele from Keio collection and removed kan marker. |
| ZD12^b^ | AH28 ∆b1304 | Transduced in allele from Keio collection and removed kan marker. |
| ZD13^b^ | AH28 ∆b1362 | Transduced in allele from Keio collection and removed kan marker. |
| ZD14^a,d^ | AH28 ∆b1818 | Transduced in allele from Keio collection and removed kan marker. |
| ZD15^d^ | AH28 ∆b2010 | Transduced in allele from Keio collection and removed kan marker. |
| ZD17^d^ | AH28 ∆b2240 | Transduced in allele from Keio collection and removed kan marker. |
| ZD18^b^ | AH28 ∆b2417 | Transduced in allele from Keio collection and removed kan marker. |
| ZD20^b^ | AH28 ∆b2587 | Transduced in allele from Keio collection and removed kan marker. |
| ZD21^c^ | AH28 ∆b2601 | Transduced in allele from Keio collection and removed kan marker. |
| ZD24^b^ | AH28 ∆b2808 | Transduced in allele from Keio collection and removed kan marker. |
| ZD25^b^ | AH28 ∆b2830 | Transduced in allele from Keio collection and removed kan marker. |
| ZD26^b^ | AH28 ∆b2905 | Transduced in allele from Keio collection and removed kan marker. |
| ZD27^b^ | AH28 ∆b2951 | Transduced in allele from Keio collection and removed kan marker. |
| ZD28^b^ | AH28 ∆b3026 | Transduced in allele from Keio collection and removed kan marker. |
| ZD29^d^ | AH28 ∆b3032 | Transduced in allele from Keio collection and removed kan marker. |
| ZD30^b^ | AH28 ∆b3089 | Transduced in allele from Keio collection and removed kan marker. |
| ZD32^c^ | AH28 ∆b3355 | Transduced in allele from Keio collection and removed kan marker. |
| ZD33^b^ | AH28 ∆b3356 | Transduced in allele from Keio collection and removed kan marker. |
| ZD35^a,d^ | AH28 ∆b3468 | Transduced in allele from Keio collection and removed kan marker. |
| ZD38^a,d^ | AH28 ∆b3542 | Transduced in allele from Keio collection and removed kan marker. |
| ZD41^b^ | AH28 ∆b3601 | Transduced in allele from Keio collection and removed kan marker. |
| ZD42^a,b^ | AH28 ∆b3609 | Transduced in allele from Keio collection and removed kan marker. |
| ZD43^c^ | AH28 ∆b3644 | Transduced in allele from Keio collection and removed kan marker. |
| ZD45^c,d^ | AH28 ∆b3751 | Transduced in allele from Keio collection and removed kan marker. |
| ZD46^c^ | AH28 ∆b3780 | Transduced in allele from Keio collection and removed kan marker. |
| ZD47^b^ | AH28 ∆b3792 | Transduced in allele from Keio collection and removed kan marker. |
| ZD48^b^ | AH28 ∆b3806 | Transduced in allele from Keio collection and removed kan marker. |
| ZD49^c^ | AH28 ∆b3888 | Transduced in allele from Keio collection and removed kan marker. |
| ZD50^b^ | AH28 ∆b3910 | Transduced in allele from Keio collection and removed kan marker. |
| ZD51^b^ | AH28 ∆b3911 | Transduced in allele from Keio collection and removed kan marker. |
| ZD52^d^ | AH28 ∆b3927 | Transduced in allele from Keio collection and removed kan marker. |
| ZD53^b^ | AH28 ∆b3963 | Transduced in allele from Keio collection and removed kan marker. |
| ZD54^d^ | AH28 ∆b4003 | Transduced in allele from Keio collection and removed kan marker. |
| ZD55^c,d^ | AH28 ∆b4015 | Transduced in allele from Keio collection and removed kan marker. |
| ZD56^b^ | AH28 ∆b4025 | Transduced in allele from Keio collection and removed kan marker. |
| ZD57^a,d^ | AH28 ∆b4081 | Transduced in allele from Keio collection and removed kan marker. |
| ZD59^c^ | AH28 ∆b4172 | Transduced in allele from Keio collection and removed kan marker. |
| ZD60^c^ | AH28 ∆b4214 | Transduced in allele from Keio collection and removed kan marker. |
| AH28 | MG1655 ∆lacZ | Girgis *et al.*, PLoS Genet 3: 1644-1660 (2007). |
