# Supplementary material for: Bacterial Adaptation through Loss of Function
Source: PLoS Genet. 2013 Jul 11;9(7):e1003617. doi: 10.1371/journal.pgen.1003617 (PMC3708842; doi:10.1371/journal.pgen.1003617)
Supplement: Table S5 — Q-values for Figure 5. a We used a Mann-Whitney test (1-sided) to compare the doubling time of each mutant with the parental strain grown in the same media. See Table S4 for a complete list of the strains tested in each media. Considering all 48 growth tests simultaneously, we chose the significance cutoff to yield a false discovery rate less than 5% (q-value<0.05). False positive calculations include mutants tested in only the first stage of the asparagine or glutamine investigation. (DOC) [file pgen.1003617.s011.doc]

| **Media** | **Strain** | **bnum** | **Gene Name** | **Mean Doubling Time (hr)** | **N** | **q-value^a^** |
| --- | --- | --- | --- | --- | --- | --- |
| Ala | ZD5 | b0688 | *pgm* | 3.10 | 6 | 0.001499 |
| Ala | ZD56 | b4025 | *pgi* | 3.28 | 7 | 0.001234 |
| Ala | ZD26 | b2905 | *gcvT* | 3.57 | 7 | 0.001234 |
| Ala | ZD18 | b2417 | *crr* | 3.75 | 6 | 0.001713 |
| Ala | ZD51 | b3911 | *cpxA* | 3.79 | 7 | 0.001645 |
| Ala | ZD1 | b0015 | *dnaJ* | 3.81 | 7 | 0.001645 |
| Ala | ZD42 | b3609 | *secB* | 3.87 | 7 | 0.001645 |
| Ala | ZD6 | b0880 | *cspD* | 4.01 | 7 | 0.002160 |
| Ala | ZD20 | b2587 | *kgtP* | 4.03 | 7 | 0.002962 |
| Ala | ZD24 | b2808 | *gcvA* | 4.09 | 7 | 0.013575 |
| Ala | ZD27 | b2951 | *yggS* | 4.14 | 7 | 0.017846 |
| Ala | ZD3 | b0209 | *yafD* | 4.18 | 7 | 0.013575 |
| Ala | ZD53 | b3963 | *fabR* | 4.18 | 7 | 0.017846 |
| Ala | AH28  (wild-type) | none |  | 4.64 | 10 |  |
| Asn | ZD8 | b0889 | *lrp* | 22.17 | 3 | 0.025974 |
| Asn | ZD17 | b2240 | *glpT* | 29.90 | 3 | 0.025974 |
| Asn | ZD29 | b3032 | *cpdA* | 30.22 | 3 | 0.025974 |
| Asn | ZD45 | b3751 | *rbsB* | 32.05 | 3 | 0.025974 |
| Asn | ZD55 | b4015 | *aceA* | 35.00 | 3 | 0.038056 |
| Asn | AH28  (wild-type) | none |  | 38.19 | 6 |  |
| Gln | ZD8 | b0889 | *lrp* | 18.45 | 6 | 0.003109 |
| Gln | ZD60 | b4214 | *cysQ* | 18.86 | 3 | 0.022222 |
| Gln | ZD59 | b4172 | *hfq* | 19.29 | 3 | 0.022222 |
| Gln | ZD32 | b3355 | *prkB* | 20.11 | 3 | 0.022222 |
| Gln | ZD49 | b3888 | *yiiD* | 20.90 | 3 | 0.022222 |
| Gln | ZD45 | b3751 | *rbsB* | 26.10 | 3 | 0.034783 |
| Gln | AH28 (wild-type) | none |  | 33.56 | 7 |  |
