# Supplementary material for: Bacterial Adaptation through Loss of Function
Source: PLoS Genet. 2013 Jul 11;9(7):e1003617. doi: 10.1371/journal.pgen.1003617 (PMC3708842; doi:10.1371/journal.pgen.1003617)
Supplement: Table S6 — Significance of overlaps between sets of differentially expressed genes. Shown are the sizes of the overlaps among the sets of genes that increased or decreased at least 2-fold on average between the mutant and the parental strain in the indicated media. The probability of an overlap of the given size or larger occurring by chance was calculated using the hypergeometric distribution. P-values were adjusted for the 24 comparisons using a Bonferroni correction. Values greater than 0.05 are not shown. (DOC) [file pgen.1003617.s012.doc]

| **M9-Ala** |  |  |  |  |  | |  |  | |  |  | |  |  |
| --- | --- | --- | --- | --- | --- | --- | --- | --- | --- | --- | --- | --- | --- | --- |
| Overlaps among set of genes whose expression increased in the mutant | | | | | | Probability of overlaps of the actual size or larger occurring by chance | | | | | | | |  |
|  | *∆dnaJ* | *∆gcvT* | *∆cpxA* | *∆pgi* |  | |  | *∆dnaJ* | | *∆gcvT* | | *∆cpxA* | *∆pgi* | |
| *∆dnaJ* | 63 |  |  |  |  | | *∆dnaJ* |  | |  | |  |  | |
| *∆gcvT* | 5 | 24 |  |  |  | | *∆gcvT* | 4.9E-4 | |  | |  |  | |
| *∆cpxA* | 3 | 5 | 148 |  |  | | *∆cpxA* |  | | 2.9E-2 | |  |  | |
| *∆pgi* | 13 | 10 | 17 | 172 |  | | *∆pgi* | 2.1E-05 | | 2.6E-7 | | 1.8E-3 |  | |
|  |  |  |  |  |  | |  |  | |  | |  |  |  |
| Overlaps among set of genes whose expression decreased in the mutant | | | | | | Probability of overlaps of the actual size or larger occurring by chance | | | | | | | |  |
|  | *∆dnaJ* | *∆gcvT* | *∆cpxA* | *∆pgi* |  | |  | *∆dnaJ* | | *∆gcvT* | | *∆cpxA* | *∆pgi* | |
| *∆dnaJ* | 84 |  |  |  |  | | *∆dnaJ* |  | |  | |  |  | |
| *∆gcvT* | 6 | 66 |  |  |  | | *∆gcvT* | 4.1E-2 | |  | |  |  | |
| *∆cpxA* | 32 | 34 | 398 |  |  | | *∆cpxA* | 1.7E-10 | | 2.4E-10 | |  |  | |
| *∆pgi* | 53 | 16 | 36 | 167 |  | | *∆pgi* | 1.9E-10 | | 5.7E-8 | | 2.2E-5 |  | |
|  |  |  |  |  |  | |  |  | |  | |  |  |  |
| **M9-Gln** |  |  |  |  |  | |  |  | |  | |  |  |  |
| Overlaps among set of genes whose expression increased in the mutant | | | | | | Probability of overlaps of the actual size or larger occurring by chance | | | | | | | |  |
|  | *∆dnaJ* | *∆lrp* | *∆hfq* | *∆cysQ* |  | |  | *∆dnaJ* | *∆lrp* | | *∆hfq* | | *∆cysQ* | |
| *∆dnaJ* | 96 |  |  |  |  | | *∆dnaJ* |  |  | |  | |  | |
| *∆lrp* | 9 | 81 |  |  |  | | *∆lrp* | 1.7E-03 |  | |  | |  | |
| *∆hfq* | 32 | 16 | 358 |  |  | | *∆hfq* | 2.1E-10 | 2.2E-2 | |  | |  | |
| *∆cysQ* | 55 | 7 | 75 | 297 |  | | *∆cysQ* | 1.7E-10 |  | | 2.0E-10 | |  | |
|  |  |  |  |  |  | |  |  |  | |  | |  |  |
| Overlaps among set of genes whose expression decreased in the mutant | | | | | | Probability of overlaps of the actual size or larger occurring by chance | | | | | | | |  |
|  | *∆dnaJ* | *∆lrp* | *∆hfq* | *∆cysQ* |  | |  | *∆dnaJ* | *∆lrp* | | *∆hfq* | | *∆cysQ* | |
| *∆dnaJ* | 146 |  |  |  |  | | *∆dnaJ* |  |  | |  | |  | |
| *∆lrp* | 71 | 175 |  |  |  | | *∆lrp* | 0 |  | |  | |  | |
| *∆hfq* | 33 | 20 | 345 |  |  | | *∆hfq* | 6.7E-7 |  | |  | |  | |
| *∆cysQ* | 50 | 33 | 235 | 422 |  | | *∆cysQ* | 1.0E-10 | 4.0E-3 | | 2.4E-10 | |  | |
